# PAHs and PCBs Affect Functionally Intercorrelated Genes in the Sea Urchin *Paracentrotus lividus* Embryos

**DOI:** 10.3390/ijms222212498

**Published:** 2021-11-19

**Authors:** Luisa Albarano, Valerio Zupo, Marco Guida, Giovanni Libralato, Davide Caramiello, Nadia Ruocco, Maria Costantini

**Affiliations:** 1 Stazione Zoologica Anton Dohrn, Department of Marine Biotechnology, Villa Comunale, 80121 Naples, Italy; luisa.albarano@szn.it (L.A.); giovanni.libralato@unina.it (G.L.); nadia.ruocco@szn.it (N.R.); 2 Department of Biology, University of Naples Federico II, Complesso di Monte Sant’Angelo, Via Cinthia 21, 80126 Naples, Italy; marguida@unina.it; 3 Stazione Zoologica Anton Dohrn, Department of Marine Biotechnology, Villa Dohrn, Punta San Pietro, 80077 Naples, Italy; vzupo@szn.it; 4 Centro Servizi Metrologici e Tecnologici Avanzati (CeSMA), Complesso Universitario di Monte Sant’Angelo, Via Cinthia 21, 80126 Naples, Italy; 5 Stazione Zoologica Anton Dohrn, Department of Research Infrastructures for Marine Biological Resources, Marine Organisms Core Facility, Villa Comunale, 80121 Naples, Italy; davide.caramiello@szn.it; 6 Stazione Zoologica Anton Dohrn, Department of Marine Biotechnology, C. da Torre Spaccata, 87071 Amendolara, Italy

**Keywords:** aromatic hydrocarbons, polychlorinated biphenyls, sea urchin

## Abstract

Polycyclic aromatic hydrocarbons (PAHs) and polychlorinated biphenyls (PCBs) represent the most common pollutants in the marine sediments. Previous investigations demonstrated short-term sublethal effects of sediments polluted with both contaminants on the sea urchin *Paracentrotus lividus* after 2 months of exposure in mesocosms. In particular, morphological malformations observed in *P. lividus* embryos deriving from adults exposed to PAHs and PCBs were explained at molecular levels by *de novo* transcriptome assembly and real-time qPCR, leading to the identification of several differentially expressed genes involved in key physiological processes. Here, we extensively explored the genes involved in the response of the sea urchin *P. lividus* to PAHs and PCBs. Firstly, 25 new genes were identified and interactomic analysis revealed that they were functionally connected among them and to several genes previously defined as molecular targets of response to the two pollutants under analysis. The expression levels of these 25 genes were followed by Real Time qPCR, showing that almost all genes analyzed were affected by PAHs and PCBs. These findings represent an important further step in defining the impacts of slight concentrations of such contaminants on sea urchins and, more in general, on marine biota, increasing our knowledge of molecular targets involved in responses to environmental stressors.

## 1. Introduction

Marine organisms are permanently exposed to multiple stressors, such as climate changes [1,2,3] and the consequential ocean acidification, deoxygenation and sea-level rise [4,5,6], natural toxic metabolites [7,8,9,10,11] and compounds deriving from human activities [12,13,14,15]. The exposure to these stressors induces marine organisms to adopt strategies against external or internal environmental factors through metabolic and/or molecular changes to maintain cellular homeostasis [16,17].

Human activities represent a major source of stress for marine organisms. Anthropic activities can change, for example, the natural compositions, rate, deposition and transport of sediment, causing a radical shift of biological responses [18]. Since sediments produce the habitat for several benthic communities, their pollution could negatively influence the fitness and survival of marine flora and fauna [19]. Sediment drains and temporarily accumulates organic and/or inorganic compounds, including polycyclic aromatic hydrocarbon (PAHs), polychlorinated biphenyls (PCB) and organotin compounds (OTCs) derived from natural and anthropogenic sources, such as industrial, commercial, agricultural and urban activities [20,21,22,23]. Following several disruptive events (bioturbation and/or dredging), marine sediments can release these accumulated pollutants facilitating the re-allocation of contaminants within the water column [20,24,25]. This resuspension affects the marine environment and consequently human health, because pollutants can accumulate in bottom feeders and magnify through marine food webs, reaching humans through fishery products [26,27].

To our knowledge, there are several studies evaluating adaptability tolerance of sea urchins to several stress conditions, such as warming and ocean acidification [28,29,30]. However, concerning anthropogenic pollutants, the long-term exposure of sea urchins to heavy metals, PAHs and PCBs did not display any tolerance or adaptation [31,32,33,34].

PAHs and PCBs are among the most toxic pollutants commonly present in the sea. PAHs consist of a group of over 100 different organic compounds, constituted of two or more fused benzene rings and pentacyclic molecules, differing in their physical and chemical properties. PAHs can be released in the marine environment both through natural events and anthropogenic activities [35,36], with a noteworthy toxicity depending on the number of benzene rings [37]. PCBs are organic compounds, mainly derived from human activities, and considered among the most dangerous pollutants because of their toxic and carcinogenic properties, vast distribution and high persistence in the environment [37,38]. Several studies reported that PCBs were able to induce harmful effects on the embryo development of sea urchins [39,40,41]. Their direct involvement in the negative impact on both marine invertebrates’ and vertebrates’ embryo development was demonstrated by toxicity assays with pure compounds [42,43,44]. For example, the toxicity model proposed by Di Toro et al. [45], relied on assays using pure hydrocarbon compounds in dissolved phase, showing that dissolved PAHs were toxic in the absence of others compounds.

However, few studies demonstrated possible negative influences of these compounds on the survival of adults and their progenies. Our recent study proved that PAHs and PCBs induced toxigenic effects on the sea urchin *Paracentrotus lividus* embryos derived from adults exposed to sub-chronic concentrations in contaminated mesocosm, using a multi-endpoint approach and de novo transcriptomic [33].

This investigation was aimed at further exploring the target genes involved in the response of the sea urchin *P. lividus* to PAHs and PCBs, and other environmental stressors. To perform this study, sea urchin plutei (48 h post-fertilization, hpf) were exposed to slight PAH- and PCB-contaminated mesocosms, also adopted in our previous experiments [33]. Firstly, an interactomic analysis was conducted on 62 genes, previously described in *P. lividus* [46,47,48,49] in order to understand the biological and functional relationships and the gene networks in which they were involved. Twenty-five new genes were identified, being functionally interconnected to 10 genes already described in previous studies [46,47,48,49]. Two networks were obtained, including most of the correlated genes, involved in stress responses, detoxification and developmental processes. Finally, the expression levels of these twenty-five genes were followed by Real Time qPCR to identify the possible gene targets affected by PAH and PCB.

## 2. Results

### 2.1. Effects of PAHs and PCBs on Gene Expression by Real Time qPCR

The pollutants concentrations (i.e., as mean value, *n* = 3) in the spiked mesocosms at the end of the experiments were 5 µg/kg, 5 µg/L and 29.1 µg/L for PAHs in sediment, water and sea urchins (i.e., gonads), respectively; while PCBs concentrations were 5 µg/kg, 5 µg/L and < 2 µg/L in sediment, water and sea urchins (i.e., gonads), respectively. PAHs and PCBs were below the relative LOD in mesocosms.

**Figure 1:**
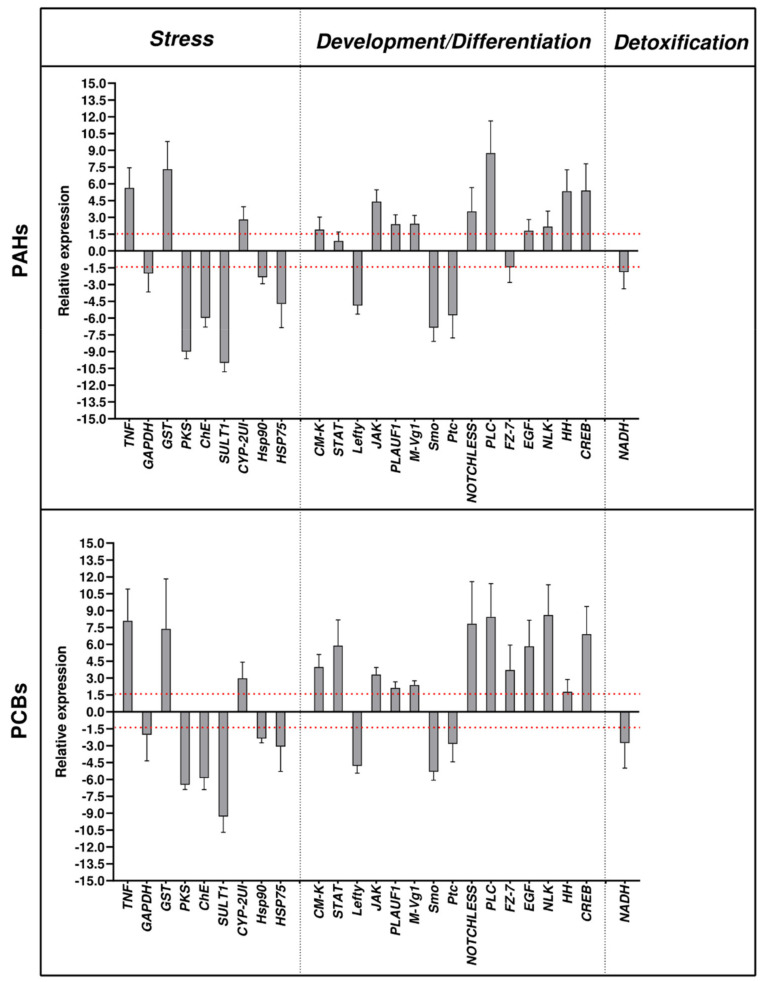
Real-time qPCR at the pluteus (48 hpf) stages of the sea urchin *P. lividus* embryos, deriving from adults exposed for 2 months to sediments contaminated with PAHs and PCBs. Data are reported as a fold difference compared with control embryos, deriving from adults reared in tanks with non-contaminated sediments (mean ± SD). Histograms show the fold-changes of 25 genes involved in three functional processes: stress response, development/differentiation and detoxification. Fold differences greater than ±1.5 (see red dotted horizontal guidelines at values of +1.5 and −1.5) were considered significant. Real-time qPCR reactions were carried out in triplicate. Statistical differences were evaluated by nonparametric Mann–Whitney test. *p*-values < 0.05 were considered significant.

PAHs and PCBs affected almost all the genes under analysis (Figure 1; fold changes are reported in Supplementary Table S1). At the pluteus stage, PAHs and PCBs had several common targets.

- Stress genes:

All nine genes were targeted by PAHs and PCBs. Both contaminants increased the expression levels of three genes (*TNF*, *GST* and *CYP-2UI*) and decreased *GAPDH*, *PKS*, *ChE*, *SULT1, hsp90* and *hsp75*.

- Genes involved in development/differentiation:

Out of 15 genes analyzed, only *STAT1* and *FZ-7* were not targeted by PAHs, whereas these two genes were up-regulated by PCBs. Common molecular targets switched on by PAHs and PCBs were *CM-K*, *JAK*, *PLAUF3*, *M-Vg1*, *NOTCHLESS*, *PLC*, *EGF*, *NLK*, *HH* and *CREB* which were up-regulated; whereas *Lefty*, *Ptc* and *Smo* were down-regulated.

- Genes involved in detoxification:

The only gene analyzed, *NADH*, was targeted by both PAHs and PCBs, which were able to decrease its expression levels.

### 2.2. Network Analysis

Twenty-five genes analyzed by Real Time-qPCR were functionally intercorrelated among them and also correlated with several genes previously isolated from *Paracentrotus lividus*. Depending on gene functions, they were ascribed to two functional networks, reporting the highest correlations among stress/detoxification and developmental genes.

**Figure 2:**
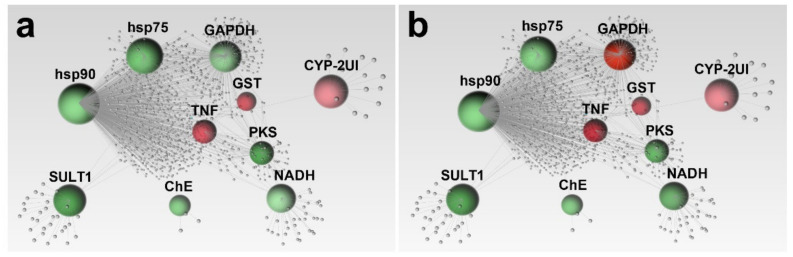
Gene networks obtained by STRING interactome analysis of ten genes involved in stress response and detoxification. Correlation confidence score cut-off of 400 was reported. Among functionally correlated genes, those with up (red) and down (green) expression affected by PAHs (**a**) and PCBs (**b**) were reported. Color shading depends on fold-change values. Gray spheres represent additional connections. The list of human orthologous genes used for Network analysis is reported in Supplementary Table S2.

Among the 10 genes involved in stress responses, *hsp90*, *hsp75* and *GAPDH* shared the largest number of connections (Figure 2).

The heat shock protein *hsp90* clearly showed a key role in several biological processes since a huge number of additional connections (grey spheres) were observed (Figure 2). *TNF* was strongly correlated to *hsp90*, *hsp75*, *GAPDH*, *GST*, *PKS* and *NADH*, while less connections have been observed for *SULT1* and *NADH*, particularly with *hasp90*, *hsp75*, *GAPDH* and *PKS* (Figure 2). *ChE* and *CYP2-UI* showed only one connection to *hsp90* through the involvement other associated genes (Figure 2).

**Figure 3:**
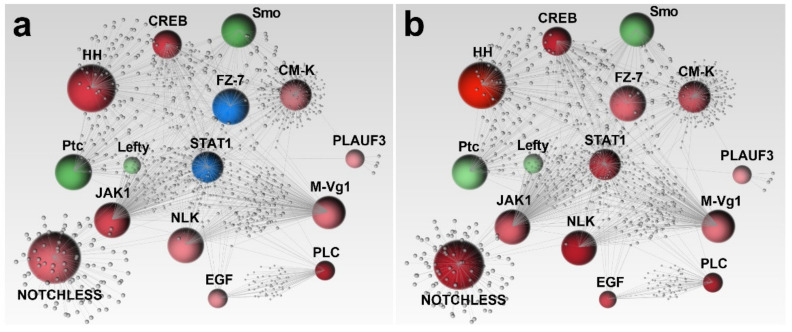
Gene network obtained by STRING interactome analysis of 15 genes involved in developmental processes. Correlation confidence score cut-off of 400 was reported. Among functionally correlated genes, those with up (red), down (green) and unchanged (blue) expression affected by PAHs (**a**) and PCBs (**b**) were reported. Color shading depends on fold-change values. Gray spheres represent additional connections. The whole list of human orthologous genes used for Network analysis is reported in Supplementary Table S2.

Among development and differentiation genes, a huge functional correlation was appreciable (Figure 3).

Several genes resulted extremely important within this biological cascade, including *HH*, *CREB*, *JAK*, *STAT1*, *M-Vg1*, *Ptc*, *Smo*, *FZ-7*, *NLK*, *NOTCHLESS* and *CM-K*, since a large number of connections was observed among them. In addition, these latter genes reported an independent association to other effectors, with *NOTCHLESS* and *CM-K* genes displaying the majority of them (Figure 3). Concerning the less correlated genes, *PLC* and *EGF* were found functionally interconnected between each other and with other genes, such as *JAK*, *STAT1* and *CAMP*, and also *Ptc* gene, in the case of *EGF*. Moreover, *PLAUF3* revealed only three functional relationships with *Smo*, *Ptc* and *STAT1* (Figure 3).

## 3. Discussion

Our results greatly expand the reach of previous investigations on genes involved in the response of the sea urchin *Paracentrotus lividus* to PAH- and PCB-contaminated sediments [33]. Previous results revealed morphological malformations in the sea urchin plutei, which were correlated to a significant alteration of the expression of several genes involved in different functional processes, such as stress response, development/differentiation, detoxification and skeletogenesis.

**Figure 4:**
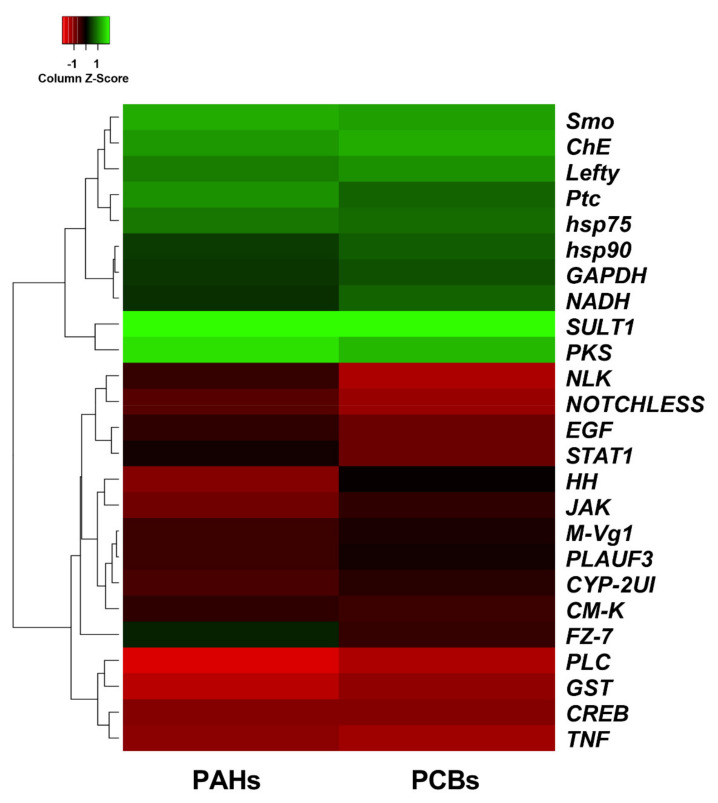
Heatmap showing the expression profiles and hierarchical clustering of 25 genes analyzed by Real Time qPCR in embryos deriving from adult *P. lividus* sea urchins exposed for 2 months to sediment contaminated with PAHs and PCBs. Color code: green, up-regulated genes with respect to the control; red, down-regulated genes with respect to the control; black, genes for which there is no variation in gene expression with respect to the control.

Here, 25 new genes involved in stress response, development/differentiation and detoxification processes were isolated in *P. lividus*. Our findings showed that PAHs and PCBs were able to switch on almost all genes under analysis (Figure 4; Supplementary Table S3).

Thus, as already reported by Albarano et al. [33], a possible correlation could be hypothesized between morphological malformations of plutei and the molecular responses exhibited. In fact, among the genes analyzed, twenty-three were altered by both pollutants, revealing comparable values of gene expression in a huge number of shared targets (Figure 1). These results could indicate that organic compounds affected some common molecular pathways, so changing physiological mechanisms in sea urchin adults, which in turn may induce abnormal offspring.

Concerning gene expression results, no significant differences were observed between PAHs and PCBs, suggesting that similar biological pathways were activated in response to PAHs and PCBs exposure. The only exception was *GAPDH*, whose expression increased in the PAHs treatment.

As for the stress response (Figure 2), both PAHs and PCBs targeted all genes analyzed in the present study. Over the last 10 years it has been shown that alteration of *ChE*, *PKS* and *GST* activity may cause morphological alterations of the spicules, similar to those shown in Figure 4 [50,51,52]. In fact, skeletal malformations and delayed larvae correlated to significant down-regulation of *PKS* gene was recently found after a 24- and 48-h exposure of *P. lividus* eggs to nickel [53]. Similarly, *CYP-2AI*, *hsp70* and *hsp90* are involved in defense against chemical stressors, and their alteration is able to impact the gastrulation process [54,55,56]. For instance, malformed embryos and up-regulation of hsp90 was found in sea urchins exposed to thermal stress and bacterial LPS, corroborating a fundamental role of this gene in the stress response [57,58,59,60]. Concerning *GAPDH*, this gene possesses key functions in the glycolytic pathway, and also roles in nuclear RNA export, membrane fusion, and DNA repair [61]. Although *GAPDH* was considered as a housekeeping gene in several organisms, including sea urchins [62,63,64], in the present study this gene was found differentially expressed after PAH and PCB exposure. The alteration of the expression level of *GAPDH* gene in sea urchin could be considered a suitable bioindicator for human health since its up-regulation was detected in cervical and ovarian tissue during cancer development [65].

As reported for the stress response network, the affected developmental and differentiation genes were almost exclusively the same for both organic compounds. A strong up-regulation of these genes was induced by sea urchin exposure to PAHs and PCBs (Figure 3). Ten functionally connected genes, *HH*, *CREB*, *NOTCHLESS*, *JAK*, *NLK*, *EGF*, *PLC*, *M-Vg1*, *PLAUF3* and *CM-K*, were up-regulated in the same biological pathway (Figure 3), thus corroborating previous results reporting several aberrations in sea urchin progenies [33]. *FZ-7* and *STAT1* were not affected by PAHs treatment (Figure 3a), indicating that the biological cascade altered by PAHs and PCBs might be slightly different. Specifically, these genes were up-regulated only by PCBs treatment. As previously mentioned for *GAPDH* gene the variation of mRNA levels might also be correlated to human diseases. In fact, the overexpression of *FZD7* was detected in 90% of tumors (most in the human hepatocellular carcinoma cells and in breast cancer cells) where causes the accumulation of β-catenin protein affecting the Wnt/β-catenin signal transduction pathway [66,67,68]. Similarly, the up-regulation of *STAT1* represents a serious risk for human health since its activation is able to down-regulate programmed cell death genes in several cancer cell lines (i.e., breast cancer and adenocarcinoma cells) [69,70,71]. As reported in Figure 3, *CREB*, *HH*, *PLAUF3*, *NLK*, *EGF*, *JAK*, *M-Vg*, *PLC* and *CM-K* were up-regulated in both treatments. Similar to our study, the expression of the transcription factor *CREB* was targeted in response to heat stress and starvation in sea urchin of the genus *Strongylocentrotus* [72,73]. The *PLC* gene is typically regulated by *CM-K*, and together they are involved in the events following fertilization and embryo development in sea urchins [74,75,76,77]. The *M-Vg* gene together with nodal is involved into left–right axis formation, regulating some down-stream effectors, including the *BMP2* gene [78]. The up-regulation of these genes could be a possible consequence of PAHs and PCBs exposures by inducing malformations in the embryo structure. The *NOTCHLESS* gene is involved in the *NOTCH-DELTA* pathway which, in turn, regulates the expression of *NLK* and *Lefty* genes [46,79,80]. This pathway has a key role during the induction and differentiation of secondary mesenchyme cells [80,81,82] and could be involved in the formation of malformed gastrulae. *Ptc* and *Smo* genes showed a down-regulation after both PAHs and PCBs treatments (Figure 3). The *Smo* and *Ptc* proteins are co-receptors of *HH* ligand and are expressed by the skeletogenic and non-skeletogenic mesoderm [83,84,85]. Probably, the defects in embryo development were caused by the perturbation of this pathway.

As described above, almost all genes were targeted by both pollutants. The sole exceptions were represented by *FZ-7* and *STAT1,* which were up-regulated by PCBs (Figure 3b). *FZ-7* receptor binding to *Wtn6* controls the β-catenin signal during the specification of the endomesoderm [86]. The *STAT1* gene together with *JAK* gene constitute the *JAK/STAT* pathway, which plays a fundamental role in the regulation of the cellular complex [87,88,89].

On the whole, molecular analyses of the aforementioned 25 genes revealed interesting results, which might justify the clear malformations affecting the apex and arms of sea urchin plutei obtained from adults exposed to PAHs and PCBs. These pollutants are released into the sea by anthropogenic activities and accumulated in marine sediments where sea urchins, typical hosts of benthic environments, could suffer for their long-lasting impacts.

In addition, an increase of knowledge on *P. lividus* gene functions has a great significance for molecular analysis on this well-established model organism. In fact, despite of its importance for the scientific community, the complete genome of the sea urchin *P. lividus* is still not fully annotated and this represents a limit in its use for molecular studies.

In the sea, when evaporation exceeds the contribution of rivers, the concentration of environmental pollutants increases with a consequent effect on organisms, biodiversity and as long-term effect on human health [90]. In fact, embryos and larvae of marine invertebrates seem to be a suitable indicator in understanding the toxicological response induced by chemical pollutants marine invertebrates, accumulating high levels of them in their tissues. Furthermore, these invertebrates have a key role in the pelagic as well in benthic food webs, representing intermediate consumers. Then, the toxicological risk faced by marine organisms and even by humans through the ingestion of contaminated edible species is concrete. Consequently, studies on the status of chemical pollutants in marine ecosystems represent an important step in evaluating possible risks on human health. On this line is also our approach with a 2-month exposure of the sea urchins to PAHs and PCBs, aiming at detecting the long-term morphological and molecular effects of these contaminants on marine invertebrates. More in general, our findings are in agreement with the idea that variations in gene expression represent an early indicator of the presence of stressful conditions in various marine environments. In fact, the identification of molecular pathways in which the targeted genes were involved represents a key step in understanding how marine organisms attempt protection against toxicants. In the case of PCBs and PAHs we found the alteration of almost the same target genes, revealing that both pollutants could activate similar biological pathways in sea urchin. This result might suggest the hypothesis that common responses of PCBs and PAHs could be also triggered in other marine invertebrates. Furthermore, this work increased the pool of genes named “defensome”, as described in Marrone et al. [91], used by marine organisms to avoid deleterious consequences and irreversible damages. In conclusion, target genes for PAHs and PCBs may be considered as possible universal biomarkers to detect the presence and the effects of key environmental pollutants impacting the physiology of marine invertebrates.

## 4. Materials and Methods

### 4.1. Experimental Conditions

Adult *Paracentrotus lividus* (seven females and three males) were reared in each of experimental tanks of a mesocosm, previously spiked with PAHs (acenaphthene (ACE), acenaphthylene (ACY), anthracene (ANT), benzo(a)anthracene (BaA), chrysene (CHR), fluoranthene (FLT), fluorine (FLR), phenanthrene (PHE), pyrene (PYR)), and PCBs (standard solution) [33]. Each mesocosm was spiked with 192 µg/L and 0.15 µg/L of PAHs and PCBs (i.e., nominal concentrations), respectively, to investigate any sub-chronic effect at concentrations below sediment threshold limit values (TLVs) (TLV_PAHs_ = 900 µg/L and TLV_PCBs_ = 8 µg/L) according to the Italian regulation D.M. 173/2016. To evaluate the compartmentalization of PAHs and PCBs in sediment and seawater, their concentrations were evaluated at three times (before (t0) and at the end (tf) of the experiment) and were measured according to Trifuoggi et al. [92]. The limit of detection and limit of quantification for PAHs and PCBs were: LOD_PAHs_ = 0.004 µg/L and LOQ_PAHs_ = 0.01 µg/L, LOD_PCBs_ = 0.002 µg/L and LOQ_PCB_ = 0.05 µg/L for the seawater samples; LOD_PAHs_ = 0.016 µg/Kg and LOQ_PAHs_ = 0.01 µg/Kg, LOD_PCBs_ = 0.03 µg/Kg and LOQ_PCBs_ = 0.01 s µg/Kg for the sediment; LOD_PCBs_ = 0.4 µg/kg w.w. and LOQ_PCBs_ = 2 µg/kg w.w. for sea urchin tissues. All details about methods for PAHs and PCBs chemical assessment were summarized in Trifuoggi et al. [92].

### 4.2. RNA Extraction and cDNA Synthesis

Adult sea urchins were collected after 2 months of exposure in PAH- and PCB-contaminated mesocosms (192 µg/L and 0.15 µg/L, respectively), and their gametes were used for in vitro fertilization [33]. Collection of embryos at the pluteus stage (about 5000 sea urchin plutei) for RNA extraction was performed according to Ruocco et al. [93]. For each sample, 1000 ng of total RNA was retrotranscribed with an iScript™ cDNA Synthesis kit (Bio-Rad, Milan, Italy), following the manufacturer’s instructions.

### 4.3. Network Analysis

Sixty-two genes [46,47,48,49] (see also Supplementary Table S4) were analyzed by NetworkAnalyst 3.0 software (https://www.networkanalyst.ca/; accessed on 8 February 2021; [94]), using STRING interactome of protein–protein interactions [95]. Human orthologous genes were used to compute the network analysis (Supplementary Table S2). The relations among genes (confidence score cut-off of 400) displaying experimental evidence were highlighted. Twenty-five mostly connected genes were then chosen and analyzed. The sequences were retrieved from the transcriptome of the sea urchin *P. lividus* deposited in the SRA database (Sequence Read Archive, available at https://www.ncbi.nlm.nih.gov/sra (accessed on 5 February 2021), accession number PRJNA495004, [96]; accession number SUB2817153, [97]; accession number SUB6701449, [33] and from NCBI (available at https://www.ncbi.nlm.nih.gov; accessed on 8 February 2021). For each gene, specific primers were designed on the nucleotide sequences (see Table 1).

PCR fragments were purified from agarose gel using the QIAquick Gel Extraction kit (Qiagen, Milan, Italy), and the specificity of the PCR product was checked by DNA sequencing. PCR products were aligned to the original sequences by MultAlin Software [98] (see Supplementary Figure S1). Five serial dilutions were set up to determine Ct values and PCR efficiencies for all primer pairs (for RT-qPCR conditions see below). Standard curves were generated for each oligonucleotide pair using Ct values versus the logarithm of each dilution factor (Supplementary Figure S2). The biological functions for 25 genes are reported in Supplementary Table S3.

### 4.4. RT-qPCR Analysis

Undiluted cDNA was used as a template in a reaction containing a 0.3 mM final concentration for each primer and 1× FastStart SYBR Green master mix (total volume of 10 μL) (Applied Biosystems, Monza, Italy). PCR amplifications were performed in a *ViiATM7 Real Time PCR System* (Applied Biosystems, Monza, Italy) thermal cycler using the following thermal profile: 95 °C for 10 min, one cycle for cDNA denaturation; 95 °C for 15 s and 60 °C for 1 min, 40 cycles for amplification; 72 °C for 5 min, one cycle for final elongation; one cycle for melting curve analysis (from 60 °C to 95 °C) to verify the presence of a single product. Each assay included a no-template control for each primer pair. To capture intra-assay variability, all *RT-qPCR* reactions were carried out in triplicate. Fluorescence was measured using the ViiATM7 software (Applied Biosystems, Monza, Italy). The relative expression ratios were calculated from quantification cycles (Cq) through an efficiency (E) corrected calculation method (Etarget ^ΔCq target (Mean Control–Mean Sample)^/Ereference ^ΔCq reference (Mean Control–Mean Sample)^) [99,100] using REST software (Version updated 2009, Relative Expression Software Tool, Weihenstephan, Germany). The expression of each gene was analyzed and internally normalized against *ubiquitin* [101] and *18S rRNA* [102,103]. Relative expression ratios above 1.5 were considered significant. Nonparametric Mann–Whitney test was applied to ∆Cq (Cq gene of interest—Cq reference) values between treated and control samples (*n* = 3). *p*-Values < 0.05 were considered significant. Statistical analyses were performed using GraphPad Prism Software (version 9.00 for Windows, GraphPad Software, La Jolla, CA, USA, www.graphpad.com, accessed on 1 February 2021).

Fold change values were represented through a Heatmap generated by Heatmapper Software (http://www.heatmapper.ca/ (accessed on 5 February 2021); [104]). Values were scaled up by column and hierarchical clustering was performed on rows by a routine adopting the Average Linkage method considering the Euclidean Distances.

### 4.5. Gene Networks

An interactomic analysis was performed by NetworkAnalyst 3.0 software [93] available at https://www.networkanalyst.ca/ (accessed on 8 March 2021), using STRING interactome of protein–protein interactions [94]. Human orthologs of selected genes were used to compute the network analysis. The most significant relations among genes (confidence score cut-off = 900) displaying experimental evidence were highlighted.

## Figures and Tables

**Table 1:** Gene Name, Acronym, Accession Numbers, Primer Names, Primer Sequences and Amplicon Lengths of PCR Fragments.

Gene Names	Acronym	Accession Number	Primer	Sequence (5′→3′)	Amplicon Lenght (bp)
*Calcium/calmodulin-dependent protein kinase type 1D*	*CM-K*	PRJNA495004	Pl_CM_F1	GTTATCCTCCATTTTACGATGAG	168
			Pl_CM_R1	GCAGATATACGTGTGAGGAAG	
*Camp-responsive element*	*CREB*	PRJNA495004	Pl_CREB_F2	GTAACTAAAGCATCTGGGAGAC	158
			Pl_CREB_R2	GGTTCAGATATTAGTGGATGC	
*Cholinesterase*	*ChE*	SUB6701449	Pl_ChE_F2	CGAGATGGCGTATGTTTTGAG	160
			Pl_ChE_R2	GACTATGTTCCCGCTGACTG	
*Citochrome P450 2UI isoform X2*	*CYP-2UI*	SUB6701449	Pl_CYP-2UI_F1	GCGCCTCTTCGTTCTATTCC	174
			Pl_CUP-2UI_R1	CGGCATAGTAGTAGACTAGC	
*Epidermal growth factor*	*EGF*	PRJNA495004	Pl_EGF_F1	CGGCGGTGTGTGTATCGATG	189
			Pl_EGF_R1	CAGTAGCCATCCTAGTGTTCC	
*Frizzled7*	*FZ-7*	PRJNA495004	Pl_FZ_F1	GATCGTGAGCGTAGCATATAC	175
			Pl_FZ_R1	CATGGTCTTTTTGGGCACTA	
*Glutathione-S-transferase*	*GST*	SUB6701449	Pl_GST_F4	GCCCGACTTACCTACTTTGC	165
			Pl_GST_R4	CTTGCAGCTCATCACTGATGG	
*Glyceraldehyde-3-phosphate dehydrogenase*	*GAPDH*	PRJNA495004	Pl_GAPDH_F1	GTACTACTTCTCATTCACCTTG	213
			Pl_GAPDH_R1	CATAGCTCTGACACCGCCAC	
*heat shock protein 75*	*hsp75*	PRJNA495004	Pl_hsp75_F2	GGACTGGTGGAACAACTATATC	173
			Pl_hsp75_R2	CGATCACCACTCTCTGTCAC	
*heat shock protein 90*	*hsp90*	SUB6701449	Pl_hsp90_F1	GGGTGTGGTAGATTCTGATG	148
			Pl_hsp90_R1	GCTCTCCATGTATTCATCAG	
*Hedgehog*	*HH*	PRJNA495004	Pl_HH_F1	GGTACATGAGGCACAAGCTAG	193
			Pl_HH_R1	CCACTTCACATCACTTGACC	
*Janus kinase*	*JAK*	XM_030985987	Pl_JAK_F2	CACCTTACCCATACTAGACAG	192
			Pl_JAK_R2	CTTGCCAGAGCCTCCGCTGAC	
*Lefty*	*Lefty*	SUB6701449	Pl_Lefty_F2	CAGTCCAGACATGGGTGGCAG	182
			Pl_Lefty_R2	CATTTCGTCGACCACCTGCTG	
*maternal Vg1*	*M-Vg1*	SUB6701449	Pl_M-Vg1_F1	GCACCTGCACCTAGAGACTC	145
			Pl_M-Vg1_R1	GCATGACCTTTTCCGGCCTG	
*NADH dehydrogenase*	*NADH*	SUB2817153	Pl_NADH_F1	GTCTCCGTCGGATAAATCAAAG	194
			Pl_NADH_R1	CCGAAAAGGAAATAACGAAGC	
*Nemo-like kinase protein*	*NLK*	AY442297	Pl_NLK_F1	CCTCTACCAGATTCTCAGAG	192
			Pl_NLK_R1	GTGACACAGTACTACCGCGC	
*Notchless protein*	*NOTCHLESS*	PRJNA495004	Pl_Notchless_F1	GGGAAGCTAAGGCATCAGAC	145
			Pl_Notchless_R1	CGATCCTCTCAAGCACTTTAG	
*Patched*	*Ptc*	SUB6701449	Pl_Ptc_F1	CGGTCGTCAGTATCATCATG	135
			Pl_Ptc_R1	GCAACCACGACTCCGTAAGC	
*Phospholipase C*	*PLC*	AJ012336	Pl_PLC_F1	GAGACATTCACAGTGCCCAC	139
			Pl_PLC_R1	CTGACCGATACCAAGCTGTAC	
*PLAUF 3 RNA-binding protein AUF1 mRNA*	*PLAUF 3*	AY682309.1	Pl_PLAUF3_F2	GGAGGATACGGCGGTGGCGG	182
			Pl_PLAUF3_R2	GTGTTGACTCCACAGGAGTG	
*Polyketide synthase*	*PKS*	SUB6701449	Pl_PKS_F1	GCTTCCTCGACCAGTCTGTC	142
			Pl_PKS_R1	CCTCCGAAGACAGTCATCTG	
*signal transducer and activator of transcription*	*STAT1*	PRJNA495004	Pl_STAT_F1	GTGTGTCAATCAGCCAGTGC	196
			Pl_STAT_R1	GTACATCATGAGCTTACCATTTC	
*Smoothened*	*Smo*	SUB6701449	Pl_Smo_F1	CACGATCCATTACGGCGTTG	217
			Pl_Smo_R1	GCCCAACTCACACCCATGAC	
*sulfotransferase 1C2-like*	*SULT1*	SUB6701449	Pl_SULT1_F2	CAGGCACTCACTGGCTCATG	140
			Pl_SULT1_R2	CTCTTCAGCTCTCGTCTTCG	
*Tumor necrosis factor alpha*	*TNF*	SUB2817153	Pl_TNF_F1	CCTGATGTGTATGCCTCTATC	144
			Pl_TNF_R1	CAAGATCCTCATGTCAGGAAG	

## Data Availability

Not applicable.

## Supplementary Materials

The following are available online at https://www.mdpi.com/article/10.3390/ijms222212498/s1, Figure S1: Alignments of PCR products to the original sequence of *GAPDH* (A), *GST* (B), *hsp75* (C), *TNF* (D), *CM-K* (E), *CREB* (F), *EGF* (G), *FZ-7* (H), *HH* (I), *NLK* (J), *NOTCHLESS* (K), *PLC* (L), *STAT1* (M), *NADH* (N), *ChE* (O), *Ptc* (P), *Smo* (Q), *hsp90* (R), *Lefty* (S), *PKS* (T), *CYP-2UI* (U), *SULT1* (V), *M-Vg1* (W), *JAK* (X), *PLAUF3* (Y) genes., Figure S2: Efficiencies (E) of *18S rRNA* (A), *Ubiquitin* (B), *ChE* (C), *CREB* (D), *CYP-2UI* (E), *GAPDH* (F), *hsp90* (G), *Lefty* (H), *NLK* (I), *NOTCHLESS* (J), *PKS* (K), *PLAUF3* (L), *Ptc* (M), *Smo* (N), *SULT1* (O) genes., Table S1: Fold changes in embryos at the pluteus stage, deriving from sea urchins exposed to PAHs and PCBs, (in red down-expressed genes; in green up-expressed genes) in comparison with the control (represented by embryos deriving from adults of sea urchins reared non-contaminated mesoscosm) at 48 hpf., Table S2: Gene name and acronym of genes from *Paracentrotus lividus* and their human orthologs., Table S3: Gene name, acronym, function and reference of 25 new genes., Table S4: Gene name, acronym and reference of 62 genes.

## References

[1] Doney S.C., Ruckelshaus M., Emmett Duffy J., Barry J.P., Chan F., English C.A., Galindo H.M., Grebmeier J.M., Hollowed A.B., Knowlton N. (2012). Climate change impacts on marine ecosystems. Ann. Rev. Mar. Sci..

[2] Gattuso J.P., Magnan A.K., Bopp L., Cheung W.W.L., Duarte C.M., Hinkel J., Mcleod E., Micheli F., Oschlies A., Williamson P. (2018). Ocean solutions to address climate change and its effects on marine ecosystems. Front. Mar. Sci..

[3] Kendall M.A., Burrows M.T., Southward A.J., Hawkins S.J. (2004). Predicting the effects of marine climate change on the invertebrate prey of the birds of rocky shores. Ibis.

[4] Fangue N.A., O’Donnell M.J., Sewell M.A., Matson P.G., MacPherson A.C., Hofmann G.E. (2010). A laboratory-based, experimental system for the study of ocean acidification effects on marine invertebrate larvae. Limnol. Oceanogr. Methods.

[5] Gattuso J.P., Magnan A., Billé R., Cheung W.W.L., Howes E.L., Joos F., Allemand D., Bopp L., Cooley S.R., Eakin C.M. (2015). Contrasting futures for ocean and society from different anthropogenic CO_2_ emissions scenarios. Science.

[6] Guinotte J.M., Fabry V.J. (2008). Ocean acidification and its potential effects on marine ecosystems. Ann. N. Y. Acad. Sci..

[7] Cutignano A., Lamari N., D’ippolito G., Manzo E., Cimino G., Fontana A. (2011). Lipoxygenase products in marine diatoms: A concise analytical method to explore the functional potential of oxylipins. J. Phycol..

[8] D’Ippolito G., Lamari N., Montresor M., Romano G., Cutignano A., Gerecht A., Cimino G., Fontana A. (2009). 15S-Lipoxygenase metabolism in the marine diatom *Pseudo-nitzschia delicatissima*. New Phytol..

[9] Fontana A., D’Ippolito G., Cutignano A., Romano G., Lamari N., Gallucci A.M., Cimino G., Miralto A., Lanora A. (2007). LOX-induced lipid peroxidation mechanism responsible for the detrimental effect of marine diatoms on zooplankton grazers. ChemBioChem.

[10] Fontana A., D’Ippolito G., Cutignano A., Miralto A., Ianora A., Romano G., Cimino G. (2007). Chemistry of oxylipin pathways in marine diatoms. Pure Appl. Chem..

[11] Miralto A., Barone G., Romano G., Poulet S.A., Ianora A., Russo G.L., Buttino I., Mazzarella G., Laablr M., Cabrini M. (1999). The insidious effect of diatoms on copepod reproduction. Nature.

[12] Anselmo H.M.R., Koerting L., Devito S., van den Berg J.H.J., Dubbeldam M., Kwadijk C., Murk A.J. (2011). Early life developmental effects of marine persistent organic pollutants on the sea urchin *Psammechinus miliaris*. Ecotoxicol. Environ. Saf..

[13] Bellas J., Saco-Álvarez L., Nieto Ó., Beiras R. (2008). Ecotoxicological evaluation of polycyclic aromatic hydrocarbons using marine invertebrate embryo-larval bioassays. Mar. Pollut. Bull..

[14] Cunningham V.L., Buzby M., Hutchinson T., Mastrocco F., Parke N., Roden N. (2006). Effects of HUMAN on Aquatic Life. Europe.

[15] Lein N.P.H., Fujii S., Tanaka S., Nozoe M., Tanaka H. (2008). Contamination of perfluorooctane sulfonate (PFOS) and perfluorooctanoate (PFOA) in surface water of the Yodo River basin (Japan). Desalination.

[16] Dutta S.M., Mustafi S.B., Raha S., Chakraborty S.K. (2018). Biomonitoring role of some cellular markers during heat stress-induced changes in highly representative fresh water mollusc, *Bellamya bengalensis*: Implication in climate change and biological adaptation. Ecotoxicol. Environ. Saf..

[17] Mizrahi T., Goldenberg S., Heller J., Arad Z. (2015). Natural variation in resistance to desiccation and heat shock protein expression in the land snail *Theba pisana* along a climatic gradient. Physiol. Biochem. Zool..

[18] Burton G.A., Johnston E.L. (2010). Assessing contaminated sediments in the context of multiple stressors. Environ. Toxicol. Chem..

[19] Yi Y., Yang Z., Zhang S. (2011). Ecological risk assessment of heavy metals in sediment and human health risk assessment of heavy metals in fishes in the middle and lower reaches of the Yangtze River basin. Environ. Pollut..

[20] Novelli A.A., Losso C., Libralato G., Tagliapietra D., Pantani C., Ghirardini A.V. (2006). Is the 1:4 elutriation ratio reliable? Ecotoxicological comparison of four different sediment:water proportions. Ecotoxicol. Environ. Saf..

[21] Libralato G., Losso C., Novelli A.A., Citron M., Della Sala S., Zanotto E., Cepak F., Ghirardini A.V. (2008). Ecotoxicological evaluation of industrial port of Venice (Italy) sediment samples after a decontamination treatment. Environ. Pollut..

[22] Lofrano G., Libralato G., Minetto D., De Gisi S., Todaro F., Conte B., Calabrò D., Quatraro L., Notarnicola M. (2016). In situ remediation of contaminated marinesediment: An overview. Environ. Sci. Pollut. Res..

[23] Pougnet F., Schäfer J., Dutruch L., Garnier C., Tessier E., Dang D.H., Lanceleur L., Mullot J.U., Lenoble V., Blanc G. (2014). Sources and historical record of tin and butyl-tin species in a Mediterranean bay (Toulon Bay, France). Environ. Sci. Pollut. Res..

[24] Mamindy-Pajany Y., Libralato G., Roméo M., Hurel C., Losso C., Ghirardini A.V., Marmier N. (2010). Ecotoxicological evaluation of Mediterranean dredged sediment ports based on elutriates with oyster embryotoxicity tests after composting process. Water Res..

[25] Nikolaou A., Kostopoulou M., Petsas A., Vagi M., Lofrano G., Meric S. (2009). Levels and toxicity of polycyclic aromatic hydrocarbons in marine sediments. TrAC Trends Anal. Chem..

[26] Liu M., Chen L., He Y., Baumann Z., Mason R.P., Shen H., Yu C., Zhang W., Zhang Q., Wang X. (2018). Impacts of farmed fish consumption and food trade on methylmercury exposure in China. Environ. Int..

[27] Zupo V., Graber G., Kamel S., Plichta V., Granitzer S., Gundacker C., Wittmann K.J. (2019). Mercury accumulation in freshwater and marine fish from the wild and from aquaculture ponds. Environ. Pollut..

[28] Evans T.G., Chan F., Menge B.A., Hofmann G.E. (2013). Transcriptomic responses to ocean acidification in larval sea urchins from a naturally variable pH environment. Mol. Ecol..

[29] Hardy N.A., Lamare M., Uthicke S., Wolfe K., Doo S., Dworjanyn S., Byrne M. (2014). Thermal tolerance of early development in tropical and temperate sea urchins: Inferences for the tropicalization of eastern Australia. Mar. Biol..

[30] Sherman E. (2015). Can sea urchins beat the heat? Sea urchins, thermal tolerance and climate change. PeerJ.

[31] Au D.W.T., Lee C.Y., Chan K.L., Wu R.S.S. (2001). Reproductive impairment of sea urchins upon chronic exposure to cadmium. Part I: Effects on gamete quality. Environ. Pollut..

[32] Au D.W.T., Reunov A.A., Wu R.S.S. (2001). Reproductive impairment of sea urchin upon chronic exposure to cadmium. Part II: Effects on sperm development. Environ. Pollut..

[33] Albarano L., Zupo V., Caramiello D., Toscanesi M., Trifuoggi M., Guida M., Libralato G., Costantini M. (2021). Sub-chronic effects of slight pah-and pcb-contaminated mesocosms in *Paracentrotus lividus* lmk: A multi-endpoint approach and de novo transcriptomic. Int. J. Mol. Sci..

[34] Xu X., Li Y., Wang Y., Wang Y. (2011). Assessment of toxic interactions of heavy metals in multi-component mixtures using sea urchin embryo-larval bioassay. Toxicol. Vitr..

[35] Bamforth S.M., Singleton I. (2005). Bioremediation of polycyclic aromatic hydrocarbons: Current knowledge and future directions. J. Chem. Technol. Biotechnol..

[36] Walker C.H., Hopkin S.P., Sibly R.M., Peakall D.B. (2001). Principles of Ecotoxicology.

[37] Perelo L.W. (2010). Review: In situ and bioremediation of organic pollutants in aquatic sediments. J. Hazard. Mater..

[38] (2000). Richard B Meagher Phytoremediation of toxic elemental and organic pollutants. Curr. Opin. Plant Biol..

[39] Pillai M.C., Vines C.A., Wikramanayake A.H., Cherr G.N. (2003). Polycyclic aromatic hydrocarbons disrupt axial development in sea urchin embryos through a β-catenin dependent pathway. Toxicology.

[40] Steevens J.A., Slattery M., Schlenk D., Aryl A., Benson W.H. (1999). Effects of ultraviolet-B light and polyaromatic hydrocarbon exposure on sea urchin development and bacterial bioluminescence. Mar. Environ. Res..

[41] Suzuki N., Ogiso S., Yachiguchi K., Kawabe K., Makino F., Toriba A., Kiyomoto M., Sekiguchi T., Tabuchi Y., Kondo T. (2015). Monohydroxylated polycyclic aromatic hydrocarbons influence spicule formation in the early development of sea urchins (*Hemicentrotus pulcherrimus*). Comp. Biochem. Physiol. Part C Toxicol. Pharmacol..

[42] Gregorin C., Albarano L., Somma E., Costantini M., Zupo V. (2021). Assessing the ecotoxicity of copper and polycyclic aromatic hydrocarbons: Comparison of effects on *Paracentrotus lividus* and *Botryllus schlosseri*, as alternative bioassay methods. Water.

[43] Carls M.G., Holland L., Larsen M., Collier T.K., Scholz N.L., Incardona J.P. (2008). Fish embryos are damaged by dissolved PAHs, not oil particles. Aquat. Toxicol..

[44] Kiparissis Y., Akhtar P., Hodson P.V., Brown R.S. (2003). Partition-controlled delivery of toxicants: A novel in vivo approach for embryo toxicity testing. Environ. Sci. Technol..

[45] Di Toro D.M., Mcgrath J.A., Hansen D.J. (2000). Technical basis for narcotic chemicals and polycyclic aromatic hydrocarbon criteria. I. Water and tissue. Environ. Toxicol. Chem..

[46] Esposito R., Ruocco N., Albarano L., Ianora A., Manfra L., Libralato G., Costantini M. (2020). Combined effects of diatom-derived oxylipins on the sea urchin *Paracentrotus lividus*. Int. J. Mol. Sci..

[47] Ruocco N., Fedele A.M., Costantini S., Romano G., Ianora A., Costantini M. (2017). New inter-correlated genes targeted by diatom-derived polyunsaturated aldehydes in the sea urchin *Paracentrotus lividus*. Ecotoxicol. Environ. Saf..

[48] Varrella S., Romano G., Ianora A., Bentley M.G., Ruocco N., Costantini M. (2014). Molecular response to toxic diatom-derived aldehydes in the sea urchin *Paracentrotus lividus*. Mar. Drugs.

[49] Varrella S., Romano G., Costantini S., Ruocco N., Ianora A., Bentley M.G., Costantini M. (2016). Toxic diatom aldehydes affect defence gene networks in sea urchins. PLoS ONE.

[50] Beeble A., Calestani C. (2012). Expression pattern of polyketide synthase-2 during sea urchin development. Gene Expr. Patterns.

[51] Cunha I., García L.M., Guilhermino L. (2005). Sea-urchin (*Paracentrotus lividus*) glutathione S-transferases and cholinesterase activities as biomarkers of environmental contamination. J. Environ. Monit..

[52] Gambardella C., Aluigi M.G., Ferrando S., Gallus L., Ramoino P., Gatti A.M., Rottigni M., Falugi C. (2013). Developmental abnormalities and changes in cholinesterase activity in sea urchin embryos and larvae from sperm exposed to engineered nanoparticles. Aquat. Toxicol..

[53] Bonaventura R., Zito F., Chiaramonte M., Costa C., Russo R. (2018). Nickel toxicity in *P. lividus* embryos: Dose dependent effects and gene expression analysis. Mar. Environ. Res..

[54] Geraci F., Pinsino A., Turturici G., Savona R., Giudice G., Sconzo G. (2004). Nickel, lead, and cadmium induce differential cellular responses in sea urchin embryos by activating the synthesis of different HSP70s. Biochem. Biophys. Res. Commun..

[55] Goldstone J.V., Hamdoun A., Cole B.J., Howard-Ashby M., Nebert D.W., Scally M., Dean M., Epel D., Hahn M.E., Stegeman J.J. (2006). The chemical defensome: Environmental sensing and response genes in the *Strongylocentrotus purpuratus* genome. Dev. Biol..

[56] Sconzo G., Amore G., Capra G., Giudice G., Cascino D., Ghersi G. (1997). Identification and characterization of a constitutive HSP75 in sea urchin embryos. Biochem. Biophys. Res. Commun..

[57] Chiaramonte M., Inguglia L., Vazzana M., Deidun A., Arizza V. (2019). Stress and immune response to bacterial LPS in the sea urchin *Paracentrous lividus* (Lamarck, 1816). Fish Shellfish Immunol..

[58] González K., Gaitán-Espitia J., Font A., Cárdenas C.A., González-Aravena M. (2016). Expression pattern of heat shock proteins during acute thermal stress in the Antarctic sea urchin, *Sterechinus neumayeri*. Rev. Chil. Hist. Nat..

[59] Runcie D.E., Garfield D.A., Babbitt C.C., Wygoda J.A., Mukherjee S., Wray G.A. (2012). Genetics of gene expression responses to temperature stress in a sea urchin gene network. Mol. Ecol..

[60] Vergara-Amado J., Silva A.X., Manzi C., Nespolo R.F., Cárdenas L. (2017). Differential expression of stress candidate genes for thermal tolerance in the sea urchin *Loxechinus albus*. J. Therm. Biol..

[61] Nicholls C., Li H., Liu J.P. (2012). GAPDH: A common enzyme with uncommon functions. Clin. Exp. Pharmacol. Physiol..

[62] Barber R.D., Harmer D.W., Coleman R.A., Clark B.J. (2005). GAPDH as a housekeeping gene: Analysis of GAPDH mRNA expression in a panel of 72 human tissues. Physiol. Genom..

[63] Lesser M.P., Carleton K.L., Böttger S.A., Barry T.M., Walker C.W. (2011). Sea urchin tube feet are photosensory organs that express a Rhabdomeric-like Opsin and PAX6. Proc. R. Soc. B Biol. Sci..

[64] Voronina E., Marzluff W.F., Wessel G.M. (2003). Cyclin B synthesis is required for sea urchin oocyte maturation. Dev. Biol..

[65] Hansen C.N., Ketabi Z., Rosenstierne M.W., Palle C., Boesen H.C., Norrild B. (2009). Expression of CPEB, GAPDH and U6snRNA in cervical and ovarian tissue during cancer development. Apmis.

[66] Merle P., De La Monte S., Kim M., Herrmann M., Tanaka S., Von Dem Bussche A., Kew M.C., Trepo C., Wands J.R. (2004). Functional consequences of frizzled-7 receptor overexpression in human hepatocellular carcinoma. Gastroenterology.

[67] Simmons G.E., Pandey S., Nedeljkovic-Kurepa A., Saxena M., Wang A., Pruitt K. (2014). Frizzled 7 expression is positively regulated by SIRT1 and β-catenin in breast cancer cells. PLoS ONE.

[68] Vincan E., Darcy P.K., Farrelly C.A., Faux M.C., Brabletz T., Ramsay R.G. (2007). Frizzled-7 dictates three-dimensional organization of colorectal cancer cell carcinoids. Oncogene.

[69] Sasidharan Nair V., Toor S.M., Ali B.R., Elkord E. (2018). Dual inhibition of STAT1 and STAT3 activation downregulates expression of PD-L1 in human breast cancer cells. Expert Opin. Ther. Targets.

[70] Guo H., Kuang S., Song Q.L., Liu M., Sun X.X., Yu Q. (2018). Cucurbitacin I inhibits STAT3, but enhances STAT1 signaling in human cancer cells in vitro through disrupting actin filaments. Acta Pharmacol. Sin..

[71] Choi E.A., Lei H., Maron D.J., Wilson J.M., Barsoum J., Fraker D.L., El-Deiry W.S., Spitz F.R. (2003). Stat1-dependent induction of tumor necrosis factor-related apoptosis-inducing ligand and the cell-surface death signaling pathway by interferon β in human cancer cells. Cancer Res..

[72] Carrier T.J., King B.L., Coffman J.A. (2015). Gene expression changes associated with the developmental plasticity of sea urchin larvae in response to food availability. Biol. Bull..

[73] Zhan Y., Li J., Sun J., Zhang W., Li Y., Cui D., Hu W., Chang Y. (2019). The Impact of Chronic Heat Stress on the Growth, Survival, Feeding, and Differential Gene Expression in the Sea Urchin Strongylocentrotus intermedius. Front. Genet..

[74] Coward K., Owen H., Poustka A.J., Hibbitt O., Tunwell R., Kubota H., Swann K., Parrington J. (2004). Cloning of a novel phospholipase C-δ isoform from pacific purple sea urchin (*Strongylocentrotus purpuratus*) gametes and its expression during early embryonic development. Biochem. Biophys. Res. Commun..

[75] Rongish B.J., Wu W., Kinsey W.H. (1999). Fertilization-induced activation of phospholipase C in the sea urchin egg. Dev. Biol..

[76] Shearer J., De Nadai C., Emily-Fenouil F., Gache C., Whitaker M., Ciapa B. (1999). Role of phospholipase Cγ at fertilization and during mitosis in sea urchin eggs and embryos. Development.

[77] Sidhu R.S., Clough R.R., Bhullar R.P. (2005). Regulation of phospholipase C-δ1 through direct interactions with the small GTPase Ral and calmodulin. J. Biol. Chem..

[78] Duboc V., Röttinger E., Besnardeau L., Lepage T. (2004). Nodal and BMP2/4 signaling organizes the oral-aboral axis of the sea urchin embryo. Dev. Cell.

[79] Royet J., Bouwmeester T., Cohen S.M. (1998). Notchless encodes a novel WD40-repeat-containing protein that modulates notch signaling activity. EMBO J..

[80] Warner J.F., Mcclay D.R. (2014). Left-right asymmetry in the Sea Urchin. Genesis.

[81] Materna S.C., Davidson E.H. (2012). A comprehensive analysis of Delta signaling in pre-gastrular sea urchin embryos. Dev. Biol..

[82] Röttinger E., Croce J., Lhomond G., Besnardeau L., Gache C., Lepage T. (2006). Nemo-like kinase (NLK) acts downstream of Notch/Delta signalling to downregulate TCF during mesoderm induction in the sea urchin embryo. Development.

[83] Ingham P.W., Taylor A.M., Nakano Y. (1991). Role of the Drosophila patched gene in positional signalling. Nature.

[84] Nachtergaele S., Whalen D.M., Mydock L.K., Zhao Z., Malinauskas T., Krishnan K., Ingham P.W., Covey D.F., Siebold C., Rohatgi R. (2013). Structure and function of the Smoothened extracellular domain in vertebrate Hedgehog signaling. Elife.

[85] Walton K.D., Warner J., Hertzler P.H., McClay D.R. (2009). Hedgehog signaling patterns mesoderm in the sea urchin. Dev. Biol..

[86] Lhomond G., McClay D.R., Gache C., Croce J.C. (2012). Frizzled1/2/7 signaling directs β-catenin nuclearisation and initiates endoderm specification in macromeres during sea urchin embryogenesis. Development.

[87] Brown S., Hu N., Hombría J.C.G. (2001). Identification of the first invertebrate interleukin JAK/STAT receptor, the Drosophila gene domeless. Curr. Biol..

[88] Darnell J.E. (1997). STATs and gene regulation. Science.

[89] Hou S.X., Zheng Z., Chen X., Perrimon N. (2002). The JAK/STAT pathway in model organisms: Emerging roles in cell movement. Dev. Cell.

[90] Chiarelli R., Roccheri M.C. (2014). Marine Invertebrates as Bioindicators of Heavy Metal Pollution. Open J. Met..

[91] Marrone V., Piscopo M., Romano G., Ianora A., Palumbo A., Costantini M. (2012). Defensome against toxic diatom aldehydes in the sea urchin Paracentrotus lividus. PLoS ONE.

[92] Trifuoggi M., Donadio C., Mangoni O., Ferrara L., Bolinesi F., Nastro R.A., Stanislao C., Toscanesi M., Di Natale G., Arienzo M. (2017). Distribution and enrichment of trace metals in surface marine sediments in the Gulf of Pozzuoli and off the coast of the brownfield metallurgical site of Ilva of Bagnoli (Campania, Italy). Mar. Pollut. Bull..

[93] Ruocco N., Costantini S., Zupo V., Romano G., Ianora A., Fontana A., Costantini M. (2017). High-quality RNA extraction from the sea urchin *Paracentrotus lividus* embryos. PLoS ONE.

[94] Zhou G., Soufan O., Ewald J., Hancock R.E.W., Basu N., Xia J. (2019). NetworkAnalyst 3.0: A visual analytics platform for comprehensive gene expression profiling and meta-analysis. Nucleic Acids Res..

[95] Szklarczyk D., Gable A.L., Lyon D., Junge A., Wyder S., Huerta-Cepas J., Simonovic M., Doncheva N.T., Morris J.H., Bork P. (2019). STRING v11: Protein-protein association networks with increased coverage, supporting functional discovery in genome-wide experimental datasets. Nucleic Acids Res..

[96] Ruocco N., Cavaccini V., Caramiello D., Ianora A., Fontana A., Zupo V., Costantini M. (2019). Noxious effects of the benthic diatoms *Cocconeis scutellum* and *Diploneis* sp. on sea urchin development: Morphological and de novo transcriptomic analysis. Harmful Algae.

[97] Ruocco N., Costantini S., Zupo V., Lauritano C., Caramiello D., Ianora A., Budillon A., Romano G., Nuzzo G., D’Ippolito G. (2018). Toxigenic effects of two benthic diatoms upon grazing activity of the sea urchin: Morphological, metabolomic and de novo transcriptomic analysis. Sci. Rep..

[98] Corpet F. (1988). Multiple sequence alignment with hierarchical clustering. Nucleic Acids Res..

[99] Pfaffl M.W., Horgan G.W., Dempfle L. (2002). Relative expression software tool ( REST © ) for group-wise comparison and statistical analysis of relative expression results in real-time PCR. Nucleic Acids Res..

[100] Pfaffl M.W. (2001). A new mathematical model for relative quantification in real-time RT—PCR. Nucleic Acids Res..

[101] Romano G., Costantini M., Buttino I., Ianora A., Palumbo A. (2011). Nitric oxide mediates the stress response induced by diatom aldehydes in the sea urchin *Paracentrotus lividus*. PLoS ONE.

[102] Pinsino A., Matranga V., Trinchella F., Roccheri M.C. (2010). Sea urchin embryos as an in vivo model for the assessment of manganese toxicity: Developmental and stress response effects. Ecotoxicology.

[103] Ragusa M.A., Costa S., Gianguzza M., Roccheri M.C., Gianguzza F. (2013). Effects of cadmium exposure on sea urchin development assessed by SSH and RT-qPCR: Metallothionein genes and their differential induction. Mol. Biol. Rep..

[104] Babicki S., Arndt D., Marcu A., Liang Y., Grant J.R., Maciejewski A., Wishart D.S. (2016). Heatmapper: Web-enabled heat mapping for all. Nucleic Acids Res..

