# Supplementary material for: PAHs and PCBs Affect Functionally Intercorrelated Genes in the Sea Urchin Paracentrotus lividus Embryos
Source: Int J Mol Sci. 2021 Nov 19;22(22):12498. doi: 10.3390/ijms222212498 (PMC8619768; doi:10.3390/ijms222212498)
Supplement: Supplementary file 1 [file ijms-22-12498-s001.zip › ijms-1445527-Supplementary.pdf]

Supplementary Table S1. Fold changes in embryos at the pluteus stage, deriving from sea urchins exposed to PAHs and PCBs, (in red down-expressed genes; in green up-expressed genes) in comparison with the control (represented by embryos deriving from adults of sea urchins reared non-contaminated mesoscosm) at 48 hpf. Fold differences greater than ± 1.5 were considered significant. The genes were grouped according the three functional classes: stress, development/differentiation and detoxification.

		PAHs	***PCBs***
Stress	*ChE*	-5.976	-5.870
	CYP-2UI	2.808	2.977
	GAPDH	-2.018	-2.028
	GST	7.307	7.372
	HSP75	-4.736	-3.083
	Hsp90	-2.337	-2.371
	PKS	-8.996	-6.475
	SULT1	-9.998	-9.287
	TNF	5.633	8.084
Development/Differentation	*CM-K*	1.917	3.980
	CREB	5.407	6.902
	EGF	1.810	5.823
	FZ-7	-1.453	3.710
	HH	5.344	1.790
	JAK	4.412	3.314
	Lefty	-4.885	-4.797
	M-Vg1	2.433	2.384
	NLK	2.182	8.600
	NOTCHLESS	3.543	7.827
	PLAUF3	2.399	2.125
	PLC	8.740	8.440
	Ptc	-5.741	-2.837
	Smo	-6.852	-5.311
	STAT1	0.898	5.886
Detoxification	*NADH*	-1.889	-2.757

Supplementary Table S2. Gene name and acronym of genes from *Paracentrotus lividus* and their human orthologs used for Network analysis. The genes included in the stress response and development/differentiation networks are highlighted in red and blue, respectively.

**Gene name**	***Paracentrotus lividus***	***Homo sapiens***
Cholinesterase	*ChE*	*BCHE*
Cytochrome P450 2UI isoform X2	*CYP-2UI*	*CYP4F12*
Glutathione-S-transferase	*GST*	*GSTK1*
Glyceraldehyde-3-phosphate dehydrogenase	*GAPDH*	*GAPDH*
Heat shock protein 75	*hsp75*	*TRAP1*
Heat shock protein 90	*hsp90*	*HSP90AA1*
NADH dehydrogenase	*NADH*	*NDUFS1*
Polyketide synthase	*PKS*	*ARAF*
Sulfotransferase 1C2-like	*SULT1*	*SULT1A1*
Tumor necrosis factor alpha	*TNF*	*TNF*
Calcium/calmodulin-dependent protein kinase	*CM-K*	*CALM1*
Camp-responsive element	*CREB*	*CREB1*
Epidermal growth factor	*EGF*	*EGF*
Frizzled7	*FZ-7*	*FZD7*
Hedgehog	*HH*	*SHH*
Janus kinase	*JAK1*	*JAK1*
Lefty	*Lefty*	*RTTN*
Maternal Vg1	*M-Vg1*	*TAB1*
Nemo-like kinase	*NLK*	*NLK*
Notchless protein	*NOTCHLESS*	*NLE1*
Patched	*Ptc*	*PTCH1*
Phospholipase C	*PLC*	*HSPG2*
PLAUF 3 RNA-binding protein AUF1 mRNA	*PLAUF3*	*SLBP*
Signal transducer and activator of transcription	*STAT1*	*STAT1*
Smoothened	*Smo*	*SMO*

Supplementary Table S3. Gene name, acronym, function and reference of new twenty-five genes.

Gene name	Acronym	Function	References
Calcium/calmodulin-dependent protein kinase type 1D	*CM-K*	Calcium-binding protein that is present in eggs and involved in the control of nuclear envelope breakdown (NEB) during mitotic division	[1,2]
Camp-responsive element	*CREB*	Transcription factor that binds certain DNA sequences, named cAMP response elements (CRE), increasing or decreasing the expression of target genes	[3]
Cholinesterase	*ChE*	Protein belonging to a group of esterases expressed in response to environmental stresses	[4,5]
Citochrome P450 2UI isoform X2	*CYP-2UI*	This gene encodes for heme-thiolate monooxygenase enzymes, which are involved in stress response	[6,7]

Epidermal growth factor	*EGF*	Growth factor that regulates various aspects of cell growth, differentiation and morphogenesis during embryo development	[8–10]
Frizzled7	*FZ-7*	Binding to Wnt6, this receptor is responsible for initiating β-catenin nuclearisation in macromeres at the 5th cleavage, which is necessary for endoderm specification	[11]
Glyceraldehyde-3-phosphate dehydrogenase	*GAPDH*	A glycolytic enzyme that plays a major role in the production of energy required for sperm cell movement	[12–14]
Glutathione-S-transferase	*GST*	This enzyme is expressed in the intestine tissue and it is involved in the response of environmental stresses	[4]
Heat shock protein 75	*hsp75*	*hsp75* was first described, together with *hsp70*, in relation to the heat shock stress. This protein is currently known to be also expressed in response to cold or heavy metals exposure, during oogenesis and embryo development of the sea urchin *P. lividus*	[15,16]

Heat shock protein 90	*hsp90*	A family of proteins that are expressed in response to stressful conditions. *Hsp90* plays important roles in embryogenesis	[17]
Hedgehog	*HH*	Protein expressed downstream to Brachyury and FoxA in the endomesoderm gene regulatory network during gastrulation that participates to the mesoderm organization	[18]
Janus kinase	*JAK*	Transcription factor that, binding to the STAT1, plays a kay role in the developmental processes	[19,20]

Lefty	*Lefty*	This protein, together with *Delta* and *Notch*, is involved in left-right establishment in sea urchins	[21]
maternal Vg1	*M-Vg1*	This gene, together with *Nodal*, is involved in ventral-dorsal establishment in sea urchins	[22]
NADH dehydrogenase	*NADH*	This enzyme is involved in the maintenance of cellular redox homeostasis for modulating numerous biological events. Changing its phosphorylation state, *NADH* modulates sperm motility	[23,24]
Nemo-like kinase protein	*NLK*	This protein, positively regulated by Delta-Notch signal, induces the specification of mesodermal cells by downregulating TCF.	[25]

Notchless protein	*NOTCHLESS*	Protein involved in the *NOTCH* pathway implicated in the differentiation of secondary mesenchyme cells, which are fated to produce mesodermal cells	[26,27]
Phospholipase C	*PLC*	An enzyme positively regulated by the *CM* signal and involved in egg activation and during embryo development in sea urchins	[28,29]
PLAUF 3 RNA-binding protein AUF1 mRNA	*PLAUF3*	This protein, binding to *H3.3 histone* 3`UTR, is probably implicated in mRNA instability	[30]

Polyketide synthase	*PKS*	The polyketide compound synthesized by PKS gene possibly has a role in the immune defense of the developing embryo	[31]
Patched	*Ptc*	Co-receptor of HH expressed within the neighboring skeletogenic and non-skeletogenic mesoderm	[18]
Smoothened	*Smo*	Developmental regulator that actives HH signaling	[32]
Signal transducer and activator of transcription	*STAT1*	Transcription factor that, binding to the *Jak* factor, plays a kay role in the developmental processes	[19,33,34]

Sulfotransferase 1C2-like	*SULT1*	Enzyme that catalyzes the bioactivation of arylamines	[35]
Tumor necrosis factor alpha	*TNF*	Protein that restricts and terminates inflammatory responses through the modulation of the ubiquitination status of central components in *NF-κB*, *IRF3* and apoptosis signaling cascades	[36]

Supplementary Table S4. Gene name, acronym and reference of 62 genes.

	Gene name	Acronym	Reference
Stress	*ADP-ribosylation factor 1*	*ARF1*	[37]
	caspase-8	*CASP8*	[38]
	Sp-Cspe3/7L	*caspase 3/7*	[39]
	Cytochrome b	*cytb*	[40]
	DNA-methyltransferase 1	*MTase*	[40]
	ERCC excision repair 3	*ERCC3*	[41]
	Glutamine synthetase	*GS*	[40]
	Glyoxylate reductase/hydroxypyruvate reductase	*GRHPR*	[37]
	Heat Shock Protein 56	*hsp56*	[40]
	Heat Shock Protein 60	*hsp60*	[40]
	Heat Shock Protein 70	*hsp70*	[40]
	Hypoxia inducible factor 1-alpha	*HIF1A*	[39]
	Nuclear factor kappalight-chain-enhancer of activated B cells	*NF-kB*	[42]
	Poly(ADP-ribose) polymerase 1	*PARP-1*	[37]
	p38 mitogen-activated protein kinase	*p38 MAPK*	[40]

	Succinate dehydrogenase	*SDH*	[37]
	Tumor protein p53	*p53*	[39]
	14-3-3 epsilon protein	*14-3-3 ε*	[40]
Development/ Differentiation	*ALG-2 interacting protein X/1*	*Alix*	[43]
-			
	Antidorsalizing morphogenetic protein 2	*ADMP2*	[37]
	Blastula protease 10	*BP10*	[40]
	Blimp	*Blimp*	[43]
	Brachyury	*Bra*	[37]
	Cadherin-associated protein (catenin) delta 2	*δ-2-catenin*	[39]
	c-Jun N-terminal kinase	*JNK*	[44]
	Delta	*Delta*	[37]
	Forkhead box protein A	*FOXA*	[44]
	Forkhead box protein G	*FoxG*	[44]
	Forkhead box protein O	*Foxo*	[44]
	Goosecoid	*Goosecoid*	[37]

Growth factor indipendent 1	*GFI1*	[44]
Hatching enzyme	*hat*	[40]
Histone H3.3	*H3.3*	[37]
Kinesin-19	*KIF19*	[37]
Nodal	*nodal*	[44]
Notch	*Notch*	[37]
One Cut Homeobox 1	*OneCut*	[44]
Smad6	*Smad6*	[37]
SRY (sex determining region Y)-box9	*sox9*	[40]
TGF beta-activated kinase	*TAK1*	[44]
Transcription factor 4	*tcf4*	[44]
Transcription factor 7	*TCF7*	[44]
Vascular endothelial growth factor	*VEGF*	[44]
Wnt5	*Wnt5*	[43]
Wnt6	*Wnt6*	[43]
Wnt8	*Wnt8*	[43]

Skeletogenesis	*Bone morphogenetic protein 5-7*	*BMP5-7*	[40]
	Jun	*C-jun*	[42]
	Nectin	*Nec*	[40]
	Pl-p16	*p16*	[45]
	Pl-p19	*p19*	[45]
	Spicule matrix protein 30	*SM30*	[40]
	Spicule matrix protein 50	*SM50*	[40]
	Univin	*uni*	[40]
Detoxification	*Catalase*	*CAT*	[43]
	Multi drug resistance protein 1	*MDR1*	[43]
	Metallothionein	*MT*	[40]
	Metallothionein 4	*MT4*	[46]
	Metallothionein 5	*MT5*	[46]
	Metallothionein 6	*MT6*	[46]
	Metallothionein 7	*MT7*	[46]

Metallothionein 8	*MT8*	[46]

Figure S1. Alignments of PCR products to the original sequence of *GAPDH* (A), *GST* (B), *hsp75* (C), *TNF* (D), *CM-K* (E), *CREB* (F), *EGF* (G), *FZ-7* (H), *HH* (I), *NLK* (J), *NOTCHLESS* (K), *PLC* (L), *STAT1* (M), *NADH* (N), *ChE* (O), *Ptc* (P), *Smo* (Q), *hsp90* (R), *Lefty* (S), *PKS* (T), *CYP-2UI* (U), *SULT1* (V), *M-Vg1* (W), *JAK* (X), *PLAUF3* (Y) genes. Primers forward and reverse were reported in blu and green, respectively.

A)

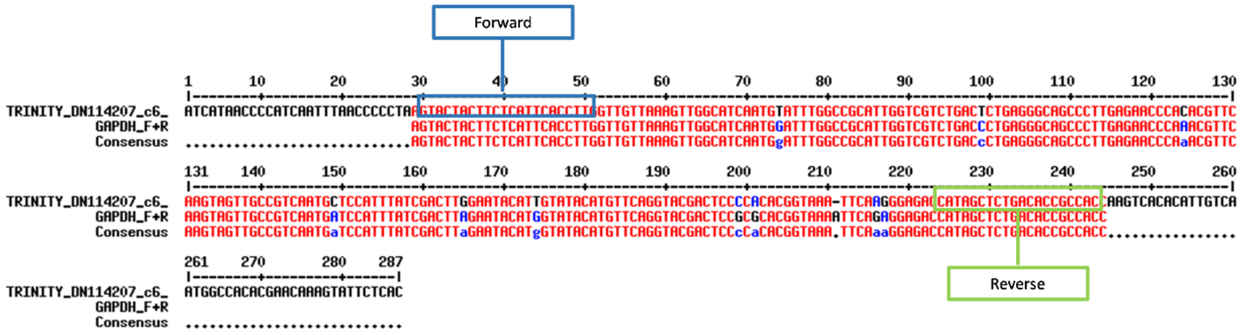

B)

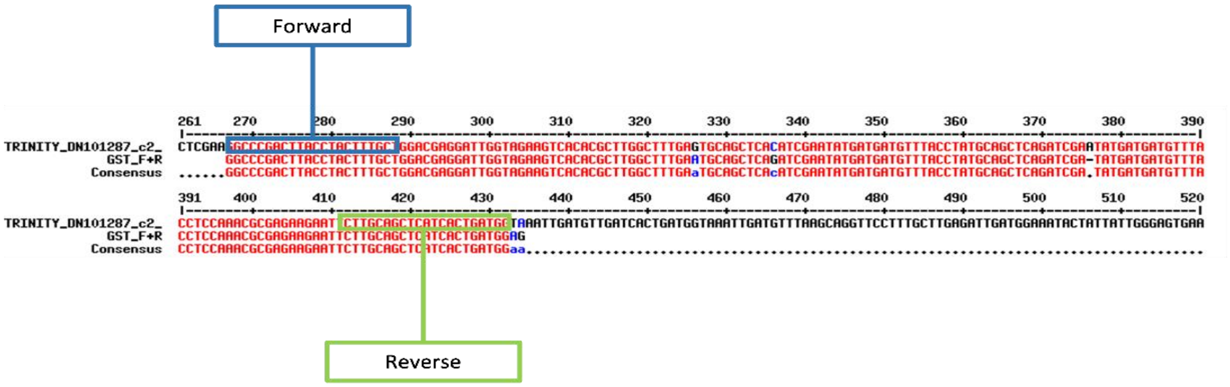

C)

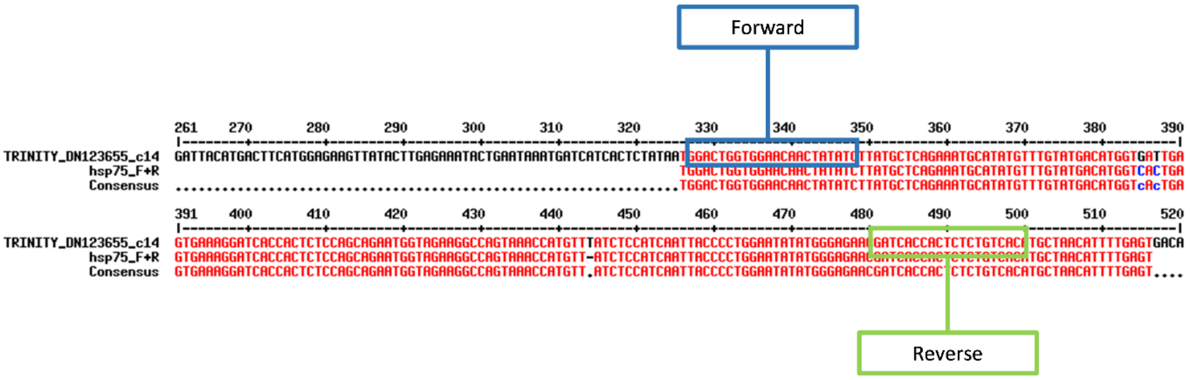

D)

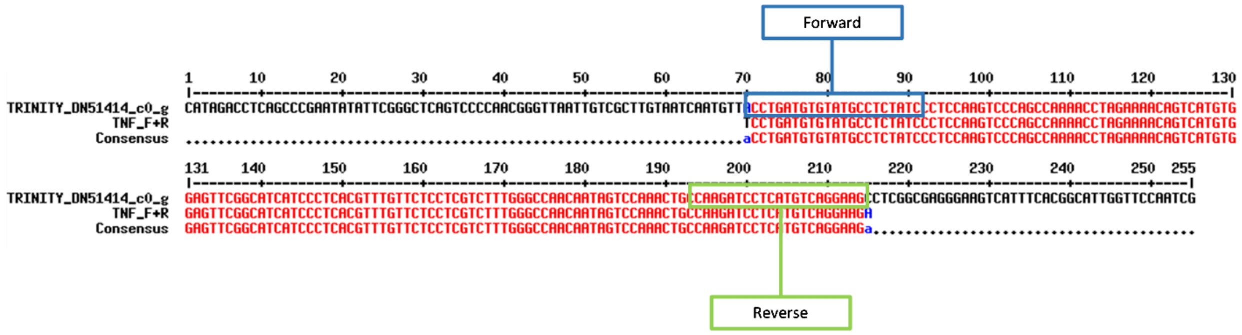
Forward
TRINITY_DN51414_c0_g
TNF_F+R
Consensus
Reverse

E)

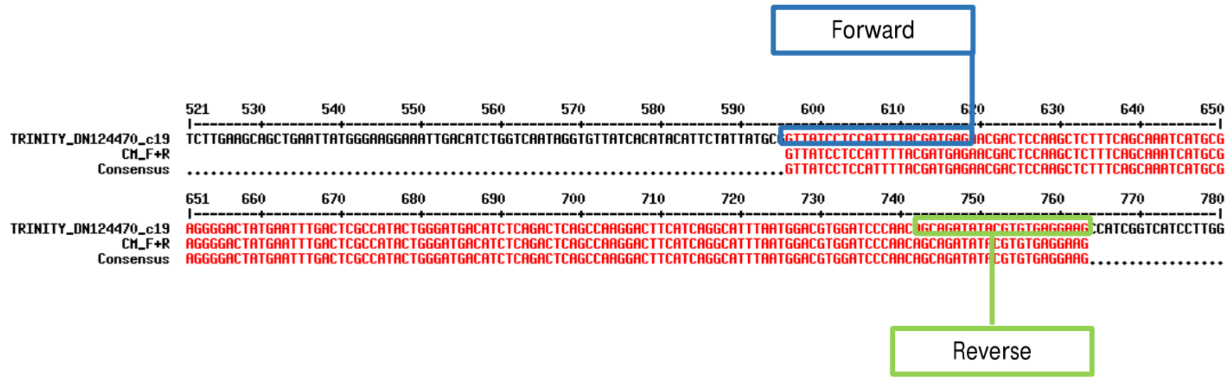
Forward
TRINITY_DN124470_c19
CM_F+R
Consensus
Reverse

F)

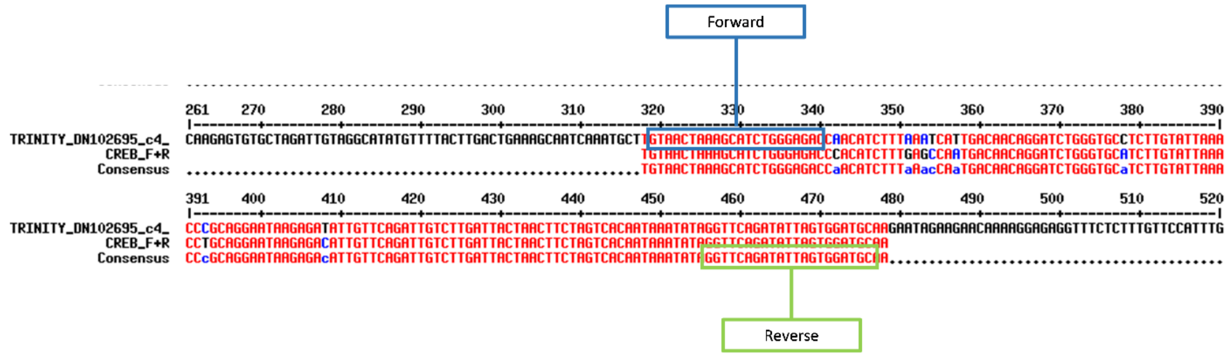
Forward
TRINITY_DN102695_c4_
CREB_F+R
Consensus
Reverse

G)

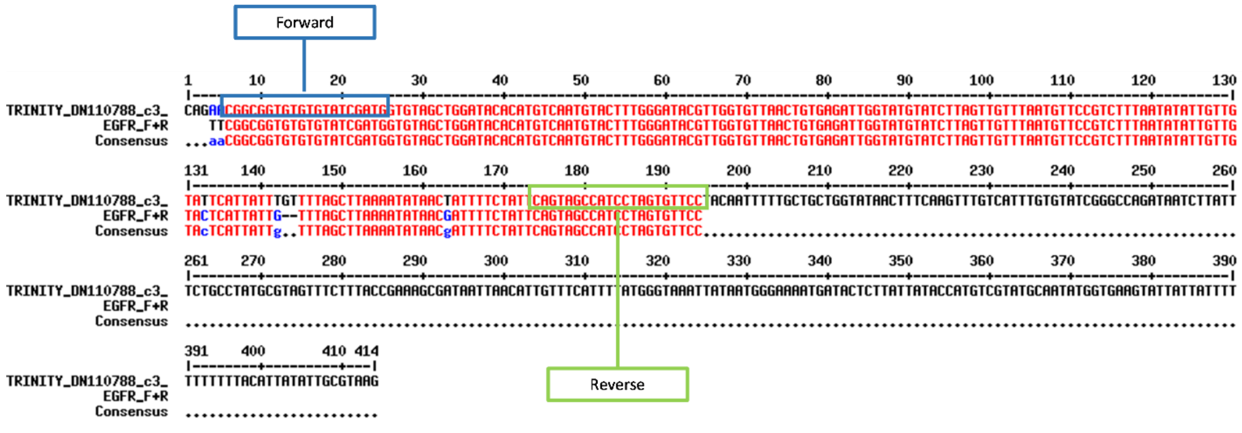
Forward
TRINITY_DN110788_c3_
EGFR_F+R
Consensus
Reverse

H)

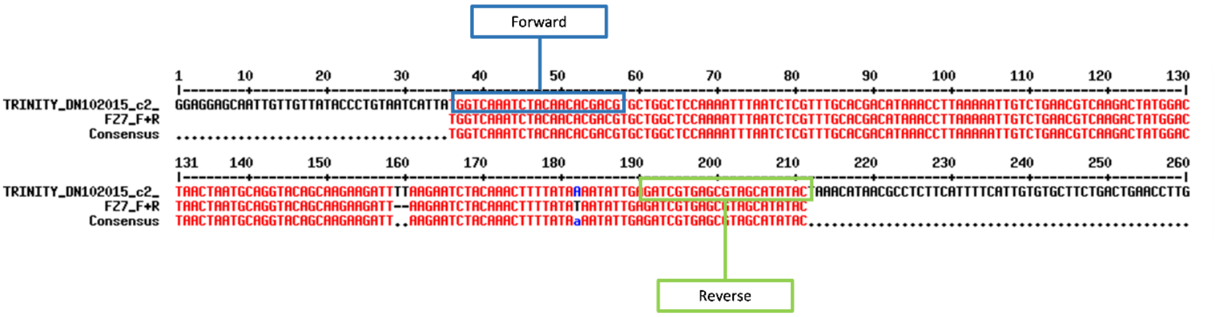
Forward
Reverse
TRINITY_DN102015_c2_
FZ7_F+R
Consensus

I)

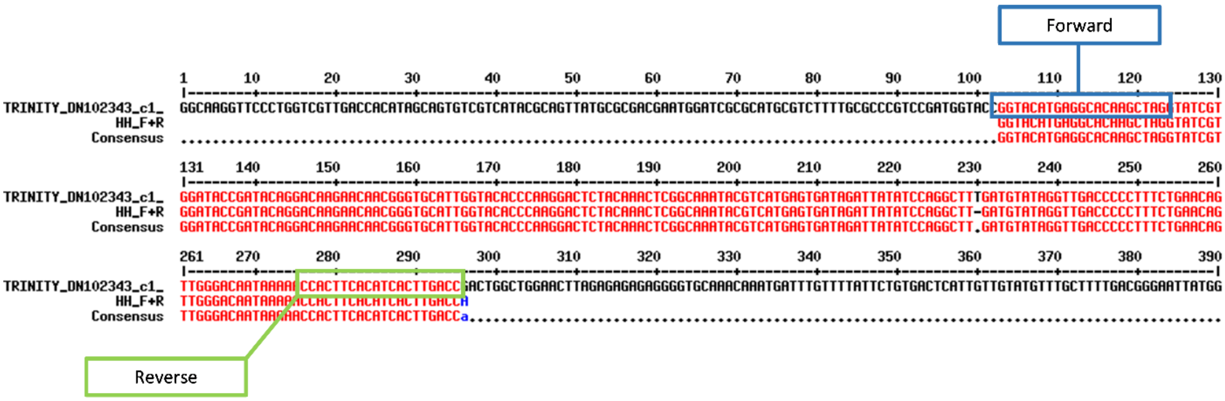
Forward
Reverse
TRINITY_DN102343_c1_
HH_F+R
Consensus

J)

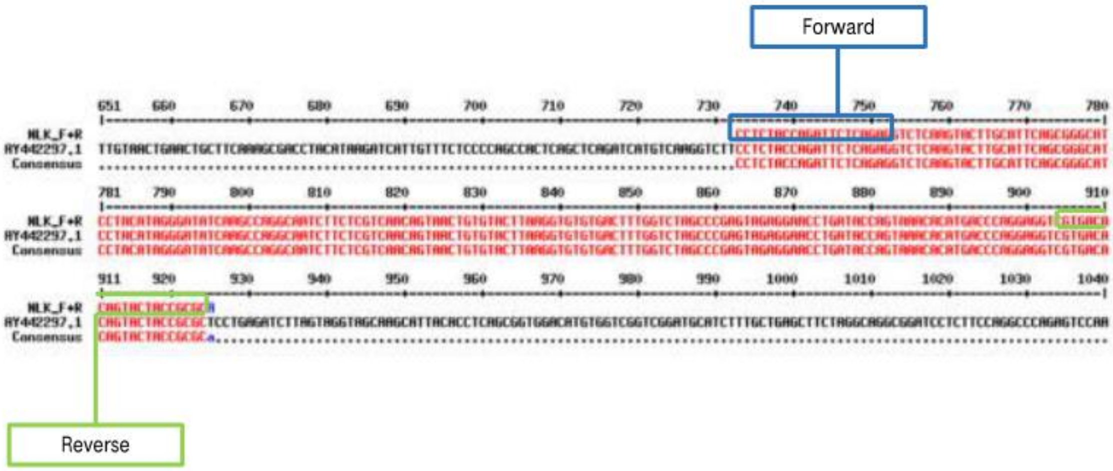
Forward
Reverse
NLK_F+R
AY442297.1
Consensus

K)

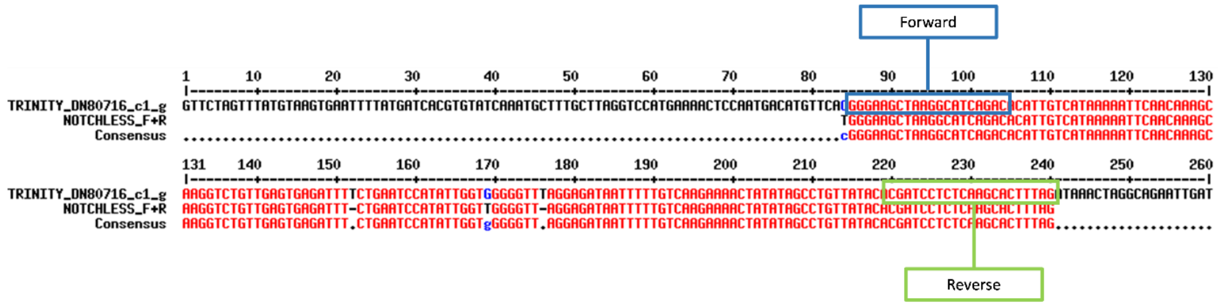
Forward
TRINITY_DN80716_c1_g
NOTCHLESS_F+R
Consensus
Reverse

L)

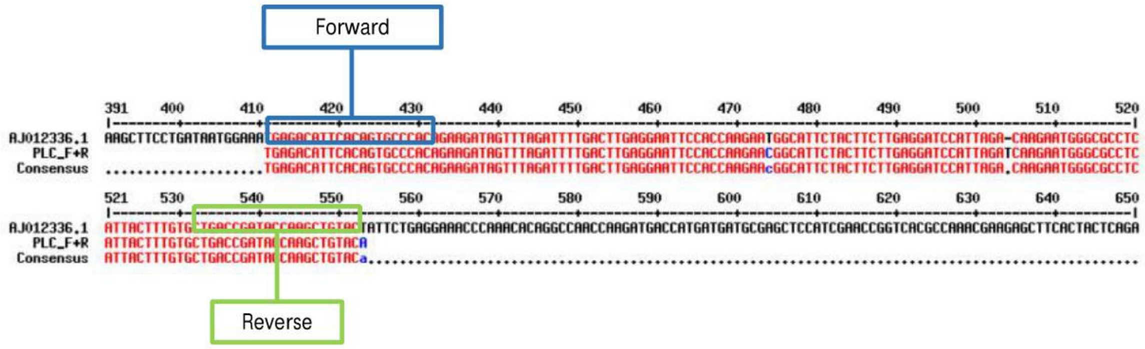
Forward
AJ012336.1
PLC_F+R
Consensus
Reverse

M)

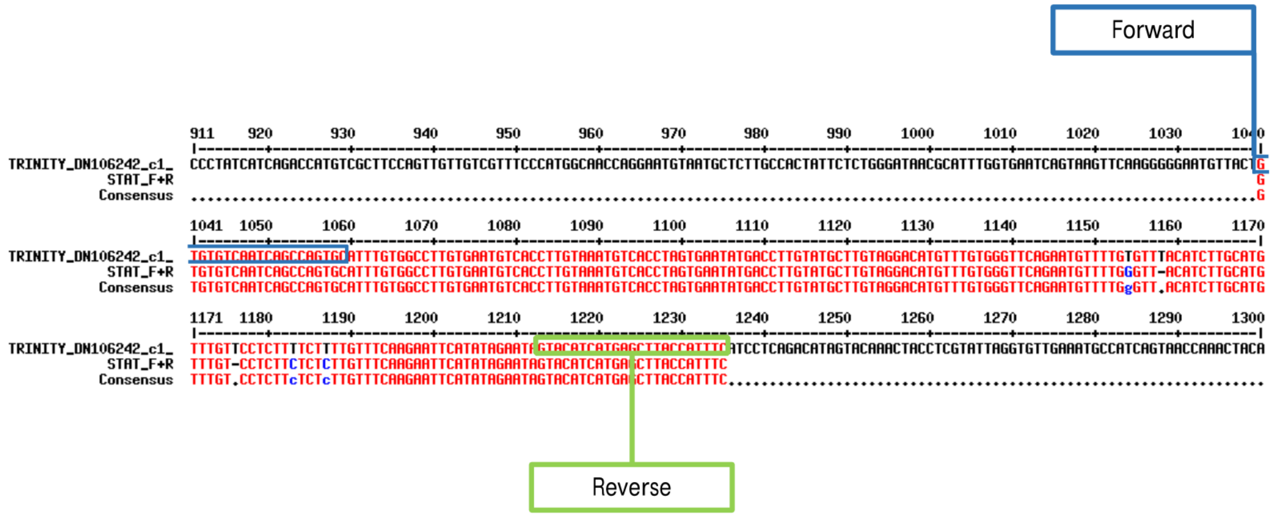
Forward
TRINITY_DN106242_c1_
STAT_F+R
Consensus
Reverse

N)

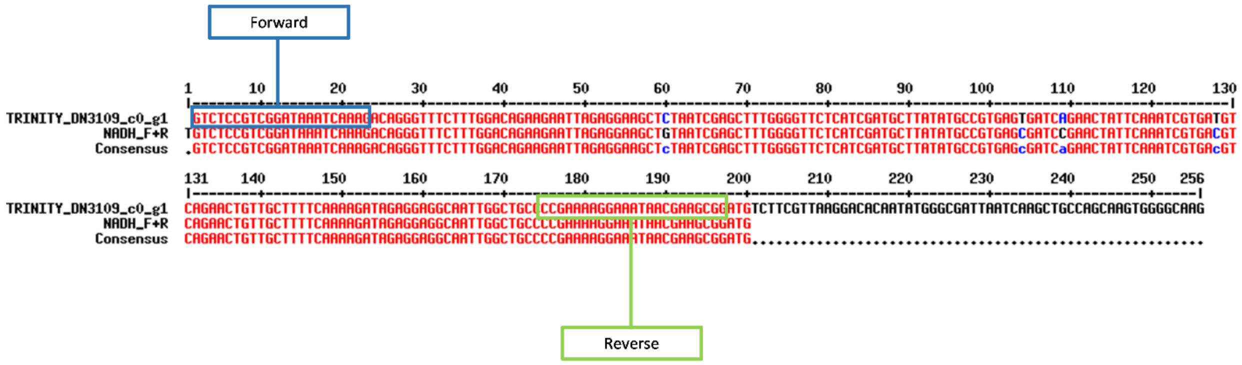
Forward
TRINITY_DN3109_c0_g1
NADH_F+R
Consensus
Reverse

O)

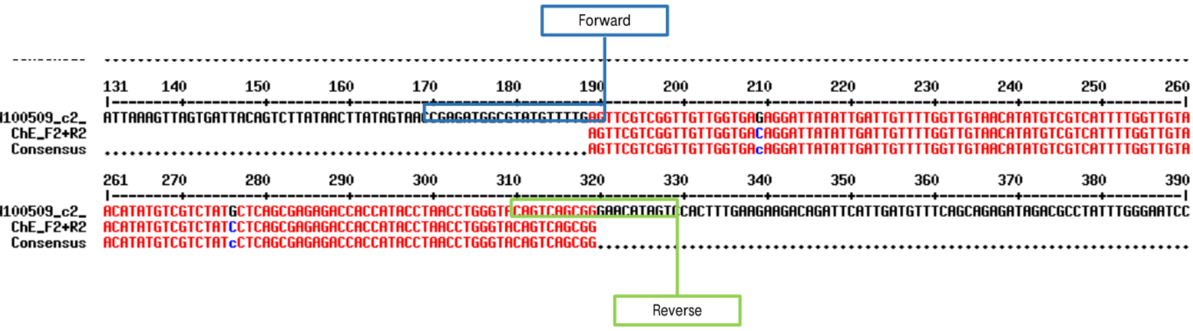
Forward
TRINITY_DN100509_c2_
ChE_F2+R2
Consensus
Reverse

P)

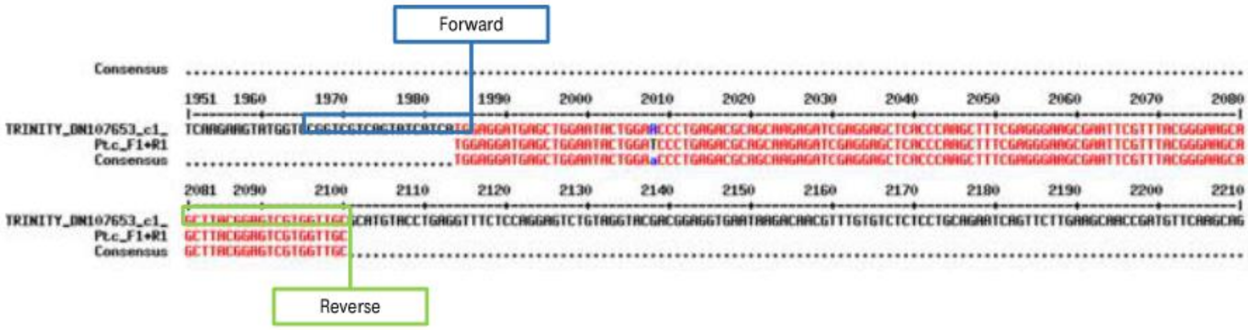
Forward
Consensus
TRINITY_DN107653_c1_
Ptc_F1+R1
Consensus
Reverse

Q)

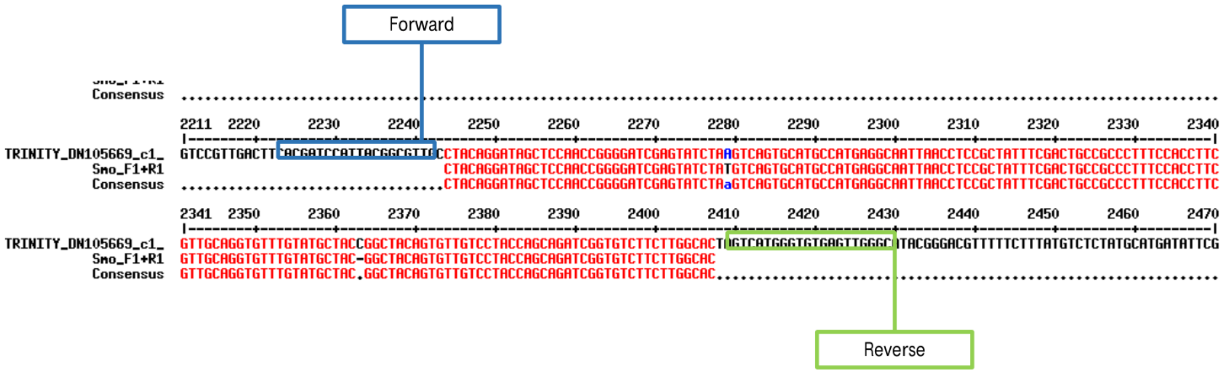
Forward
TRINITY_DN105669_c1_
Smo_F1+R1
Consensus
Reverse

R)

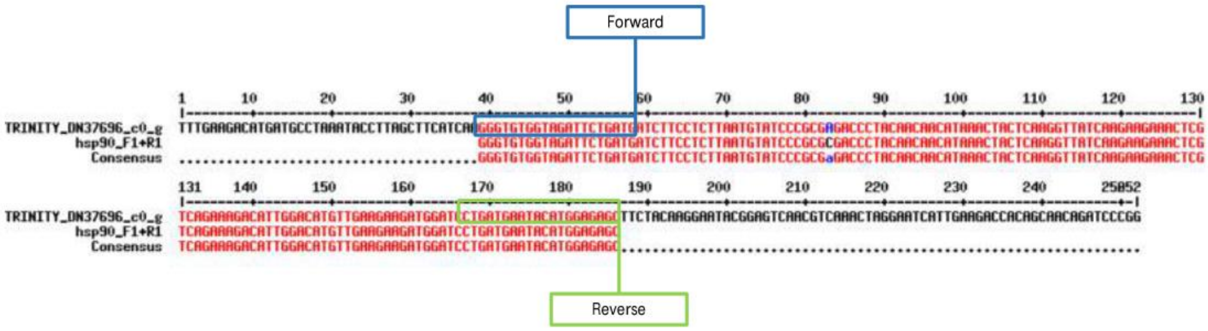
Forward
TRINITY_DN37696_c0_g
hsp90_F1+R1
Consensus
Reverse

S)

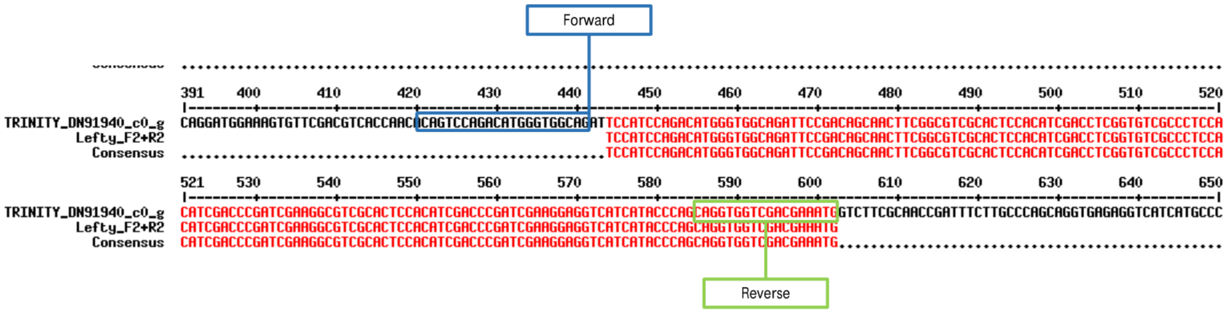
Forward
TRINITY_DN91940_c0_g
Lefty_F2+R2
Consensus
Reverse

T)

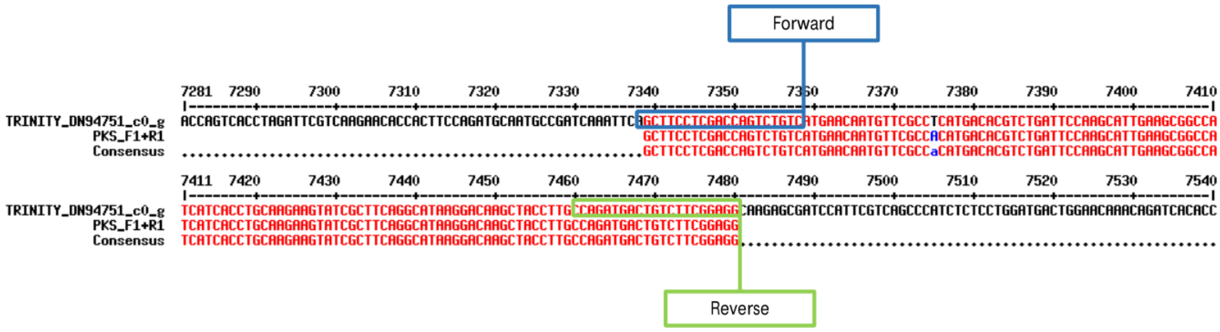
Forward
TRINITY_DN94751_c0_g
PKS_F1+R1
Consensus
Reverse

U)

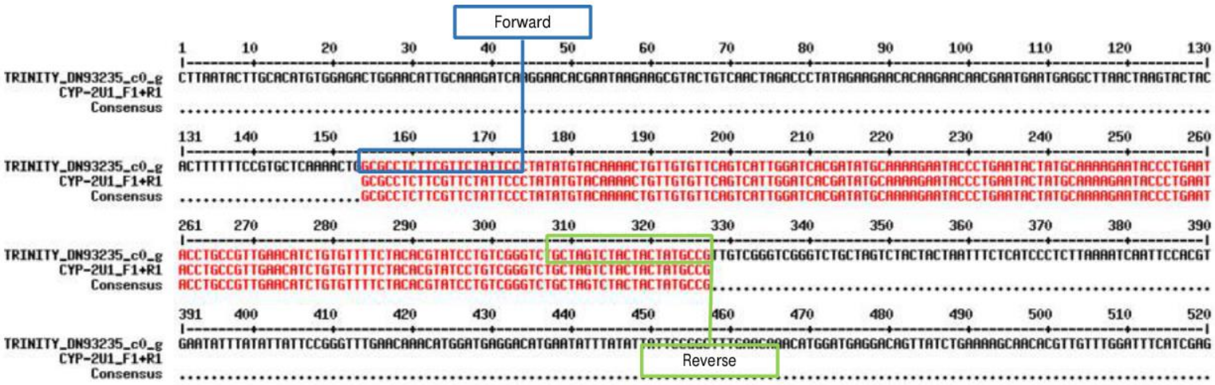
Forward
Reverse
TRINITY_DN93235_c0_g
CYP-2U1_F1+R1
Consensus

V)

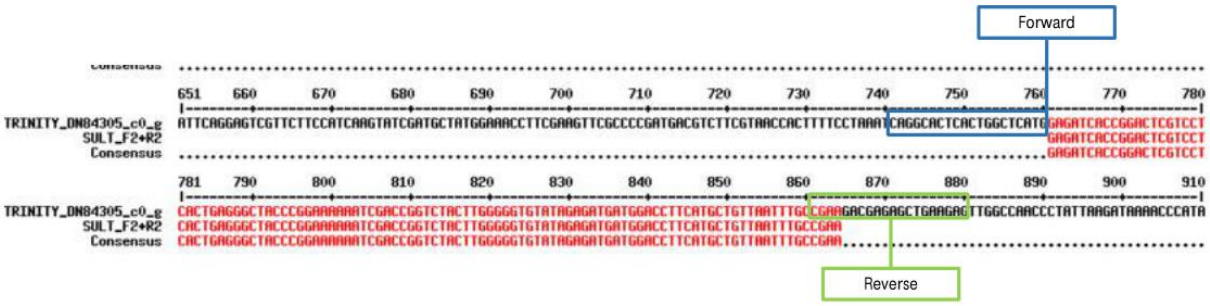
Forward
Reverse
TRINITY_DN84305_c0_g
SULT_F2+R2
Consensus

W)

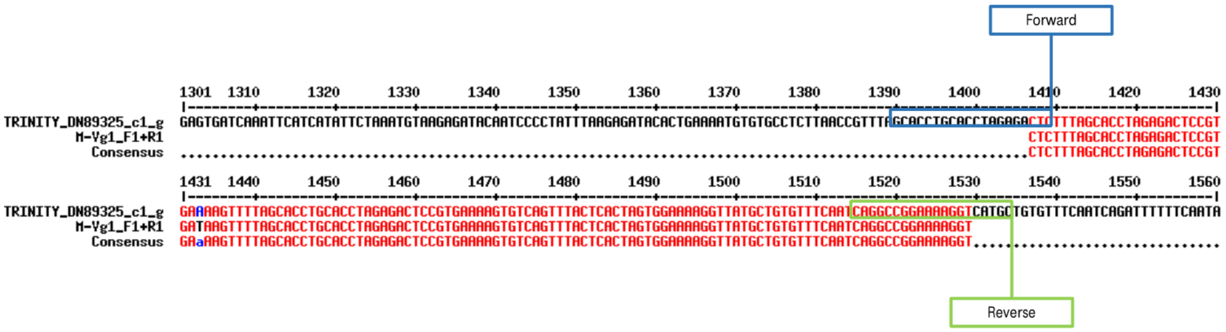
Forward
TRINITY_DN89325_c1_g
M-Vg1_F1+R1
Consensus
Reverse

X)

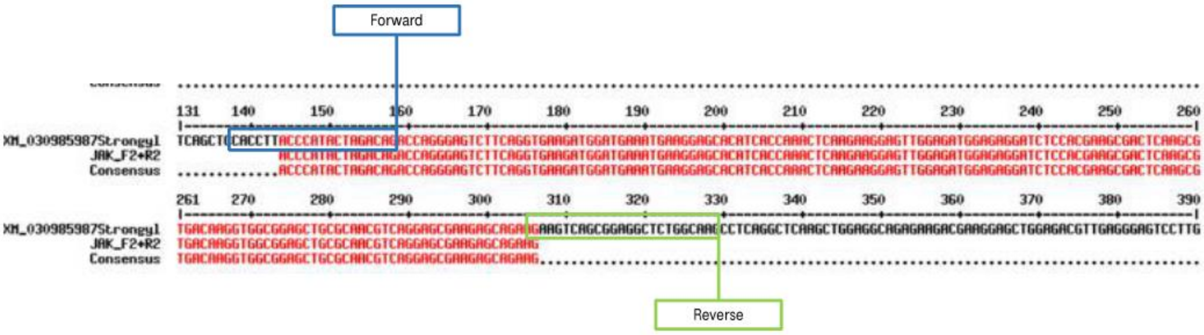
Forward
XM_030985987Strongyl
JAK_F2+R2
Consensus
Reverse

Y)

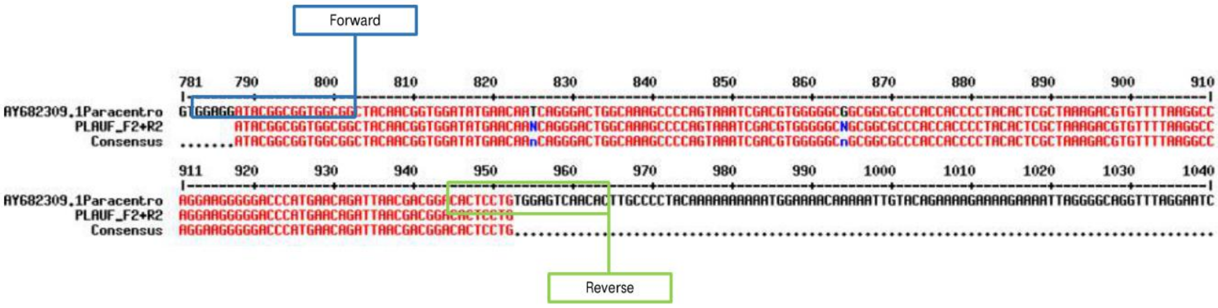
Forward
AY682309.1Paracentro
PLAUF_F2+R2
Consensus
Reverse

Figure S2. Efficiencies (E) of *18S rRNA* (A), *Ubiquitin* (B), *ChE* (C), *CREB* (D), *CYP-2UI* (E), *GAPDH* (F), *hsp90* (G), *Lefty* (H), *NLK* (I), *NOTCHLESS* (J), *PKS* (K), *PLAUF3* (L), *Ptc* (M), *Smo* (N), *SULT1* (O) genes. For those genes whose expression was detected significantly low, the efficiency was not measured.

A)

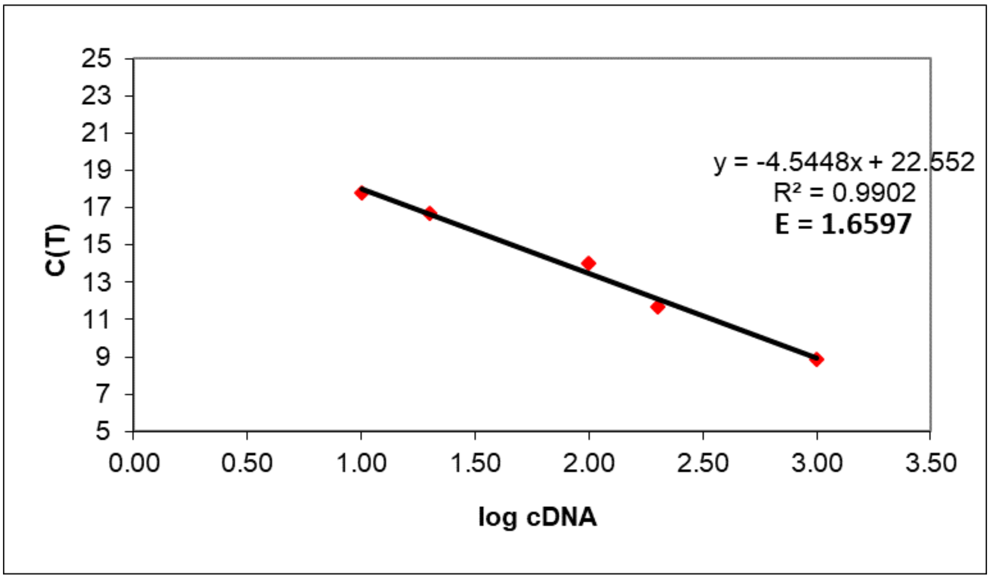

B)

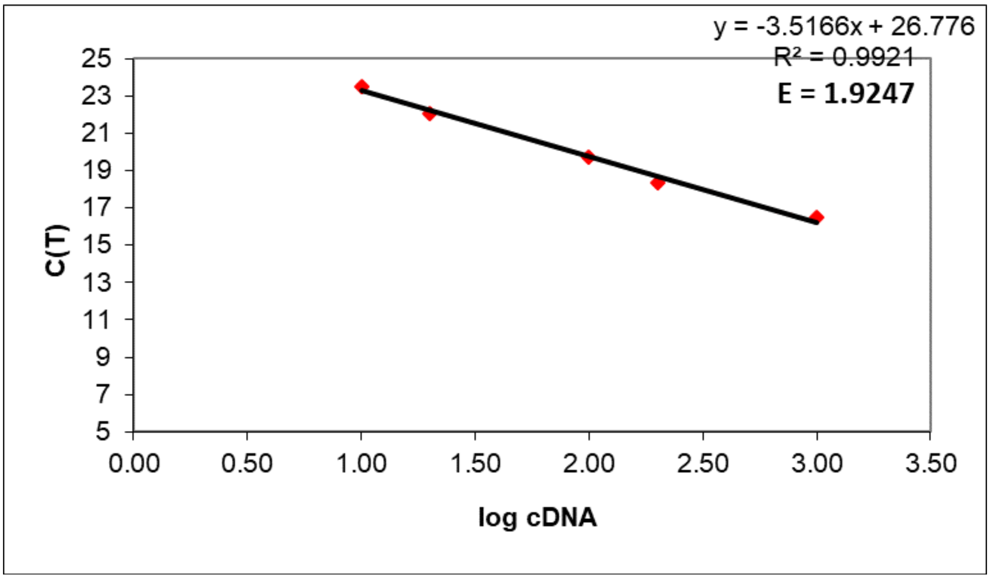

C)

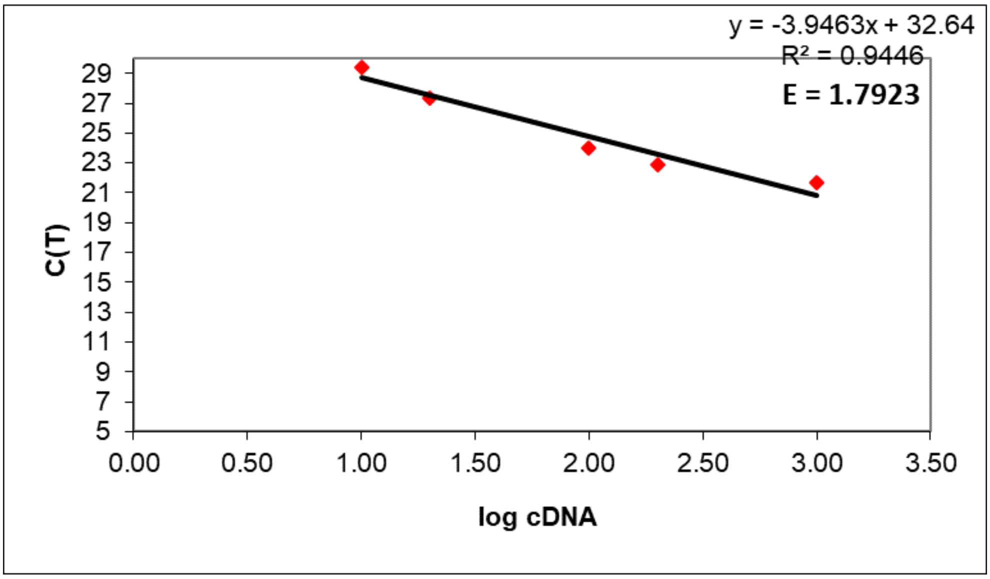
y = -3.9463x + 32.64
R² = 0.9446
E = 1.7923
C(T)
29
27
25
23
21
19
17
15
13
11
9
7
5
0.00
0.50
1.00
1.50
2.00
2.50
3.00
3.50
log cDNA

D)

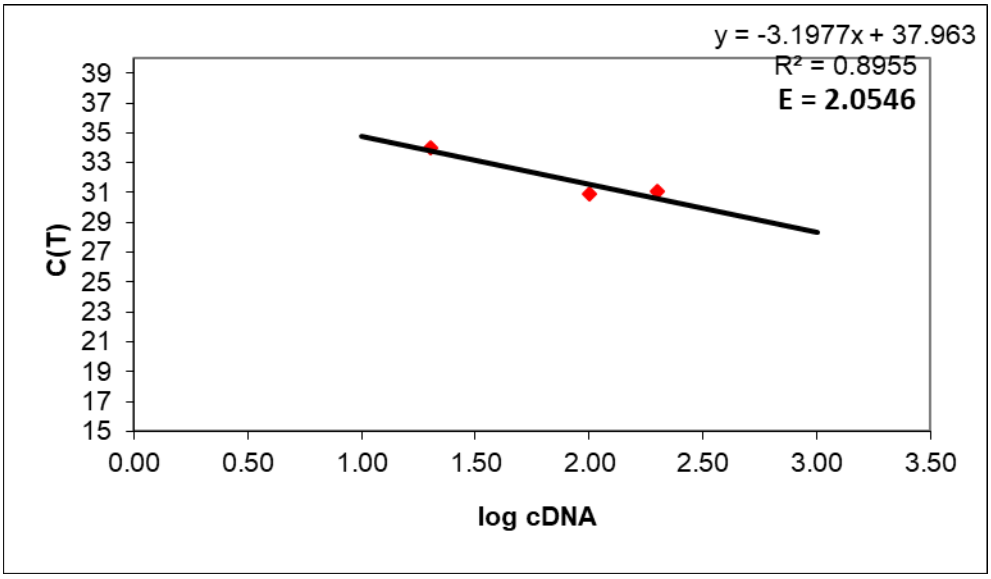
y = -3.1977x + 37.963
R² = 0.8955
E = 2.0546
C(T)
39
37
35
33
31
29
27
25
23
21
19
17
15
0.00
0.50
1.00
1.50
2.00
2.50
3.00
3.50
log cDNA

E)

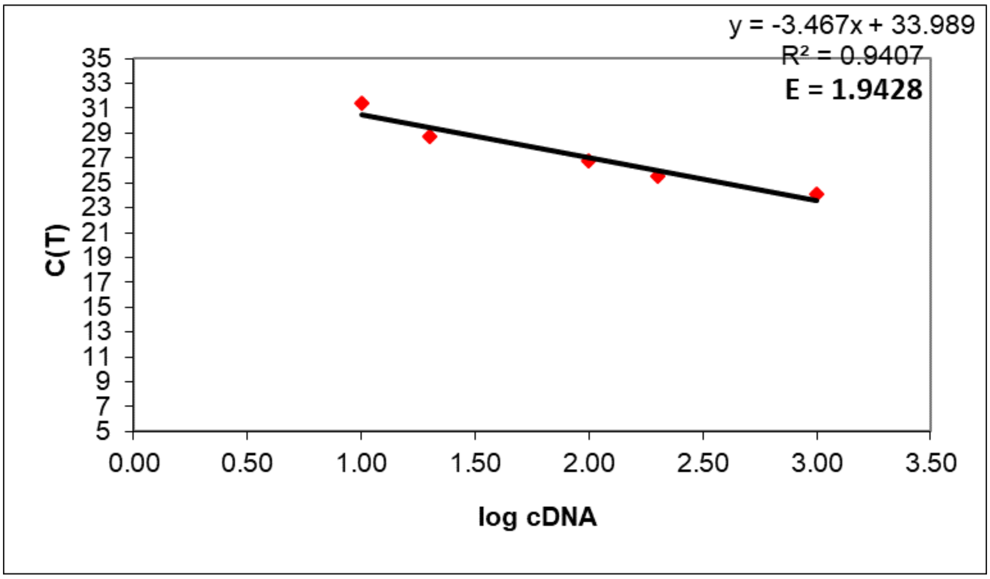
y = -3.467x + 33.989
R² = 0.9407
E = 1.9428
C(T)
35
33
31
29
27
25
23
21
19
17
15
13
11
9
7
5
0.00
0.50
1.00
1.50
2.00
2.50
3.00
3.50
log cDNA

F)

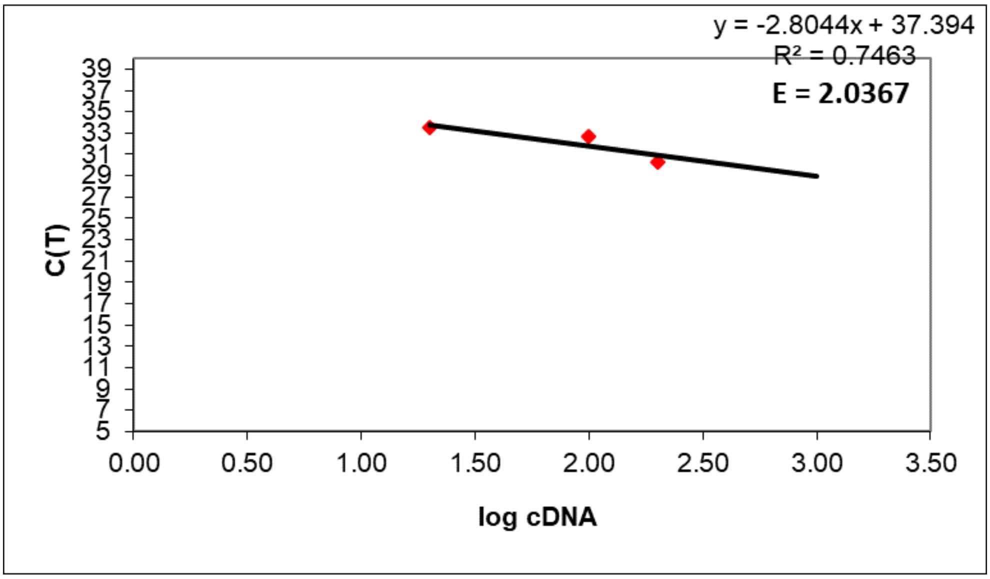
y = -2.8044x + 37.394
R² = 0.7463
E = 2.0367
C(T)
39
37
35
33
31
29
27
25
23
21
19
17
15
13
11
9
7
5
0.00
0.50
1.00
1.50
2.00
2.50
3.00
3.50
log cDNA

G)

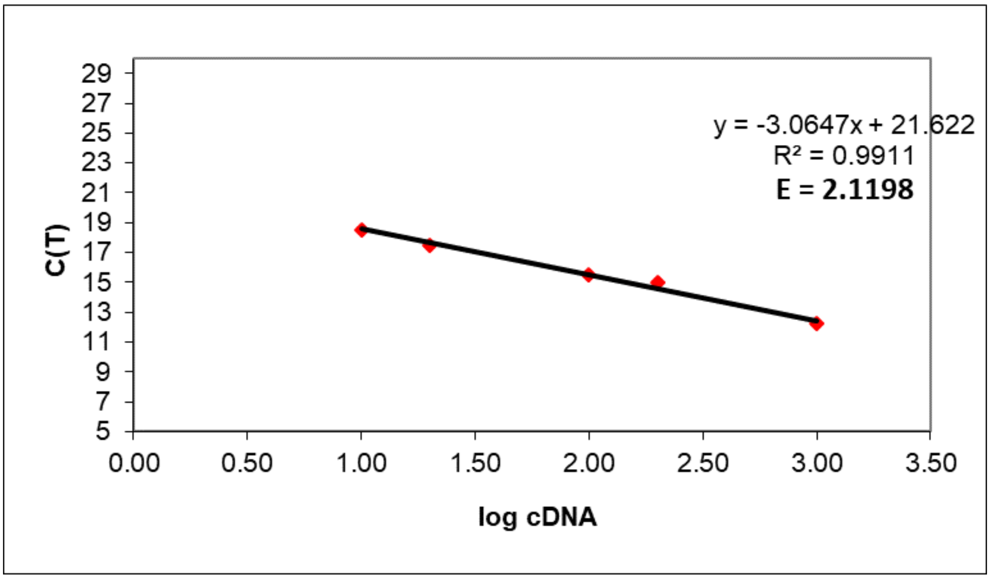
y = -3.0647x + 21.622
R² = 0.9911
E = 2.1198
C(T)
29
27
25
23
21
19
17
15
13
11
9
7
5
0.00
0.50
1.00
1.50
2.00
2.50
3.00
3.50
log cDNA

H)

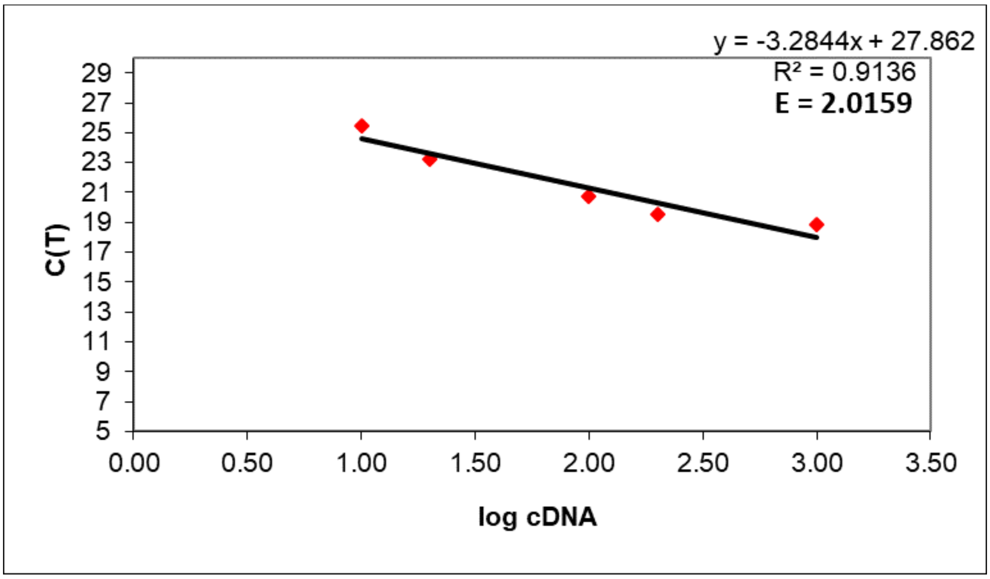
y = -3.2844x + 27.862
R² = 0.9136
E = 2.0159
C(T)
29
27
25
23
21
19
17
15
13
11
9
7
5
0.00
0.50
1.00
1.50
2.00
2.50
3.00
3.50
log cDNA

I)

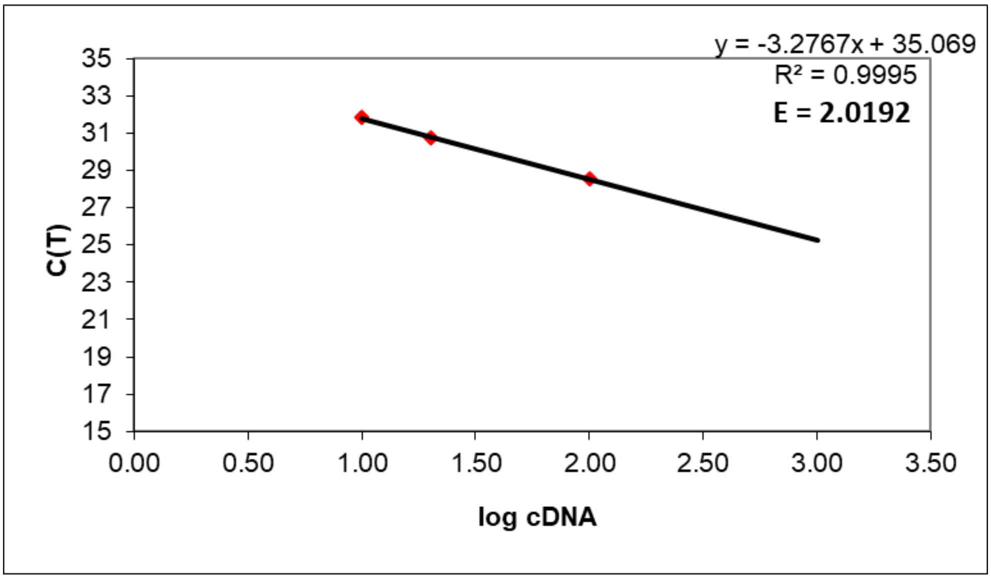
y = -3.2767x + 35.069
R² = 0.9995
E = 2.0192
C(T)
35
33
31
29
27
25
23
21
19
17
15
0.00
0.50
1.00
1.50
2.00
2.50
3.00
3.50
log cDNA

J)

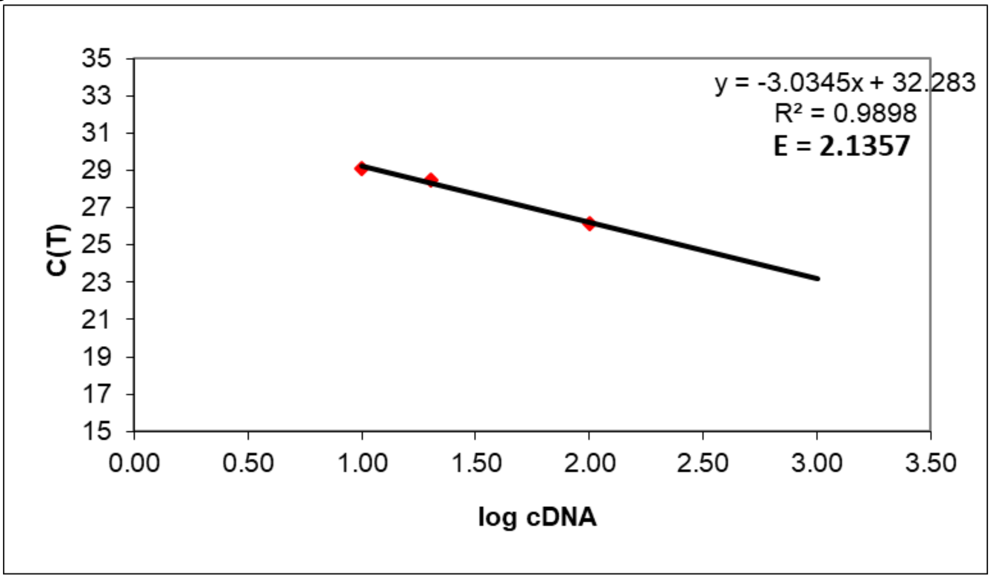
y = -3.0345x + 32.283
R² = 0.9898
E = 2.1357
C(T)
35
33
31
29
27
25
23
21
19
17
15
0.00
0.50
1.00
1.50
2.00
2.50
3.00
3.50
log cDNA

K)

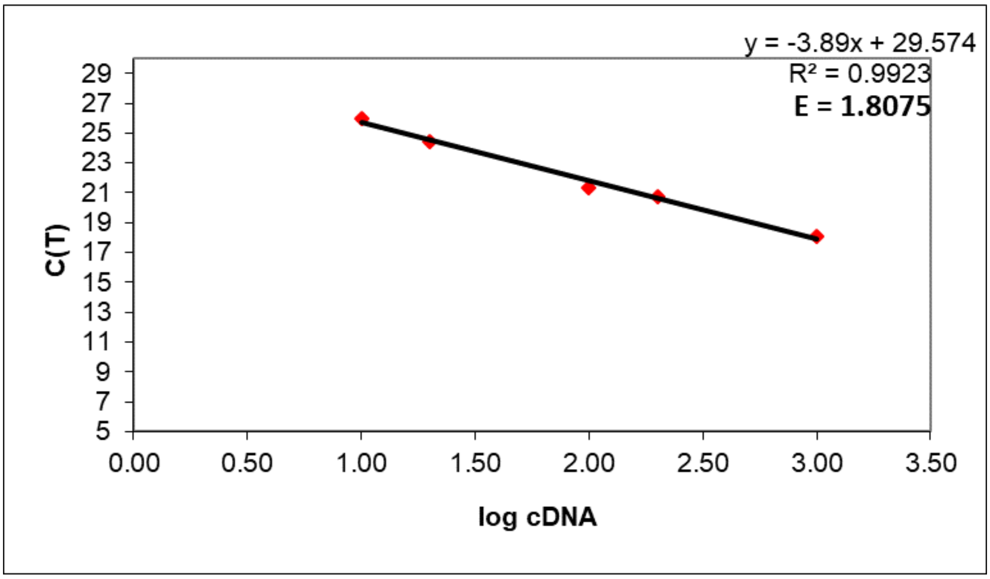
y = -3.89x + 29.574
R² = 0.9923
E = 1.8075
C(T)
log cDNA

L)

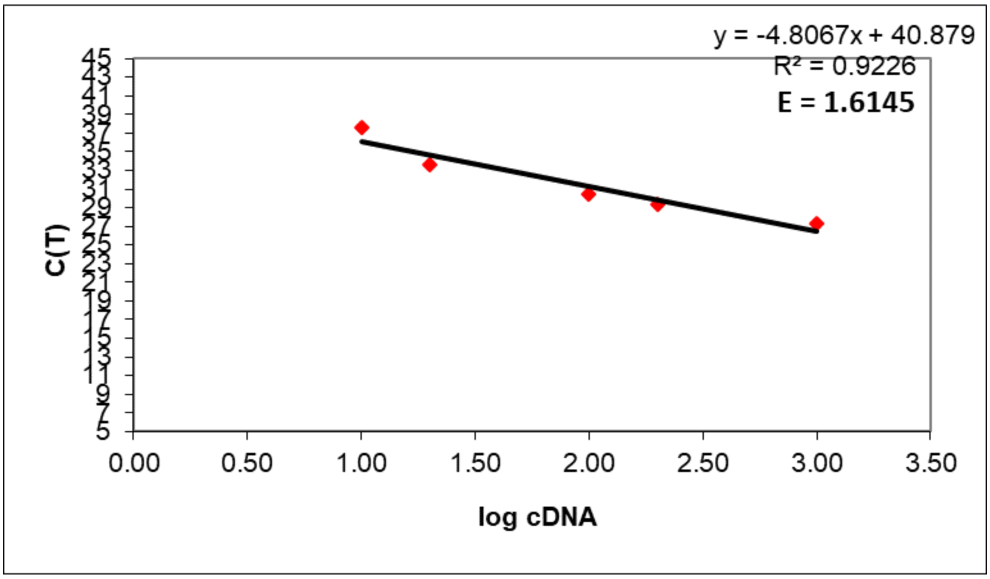
y = -4.8067x + 40.879
R² = 0.9226
E = 1.6145
C(T)
log cDNA

M)

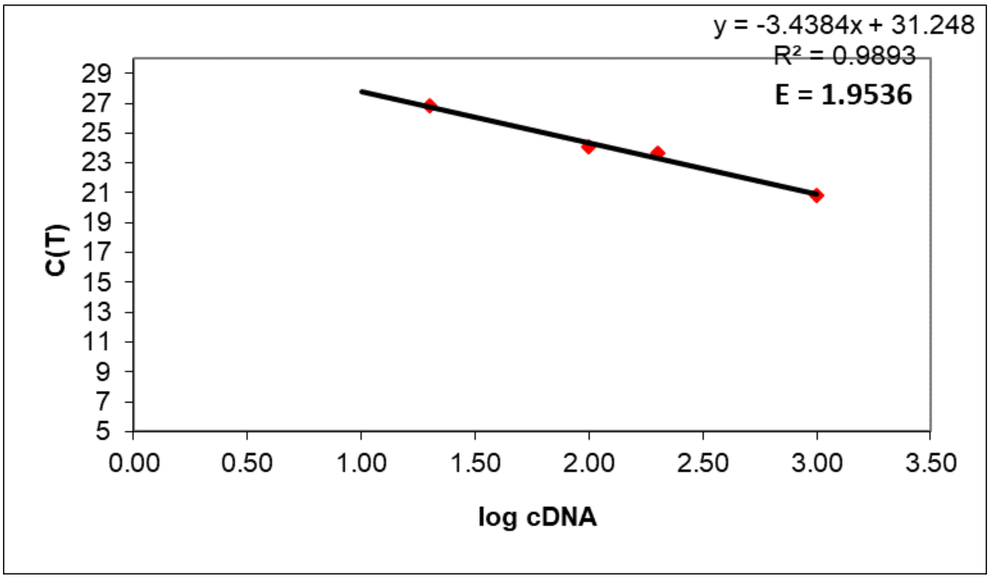
y = -3.4384x + 31.248
R² = 0.9893
E = 1.9536
C(T)
29
27
25
23
21
19
17
15
13
11
9
7
5
0.00
0.50
1.00
1.50
2.00
2.50
3.00
3.50
log cDNA

N)

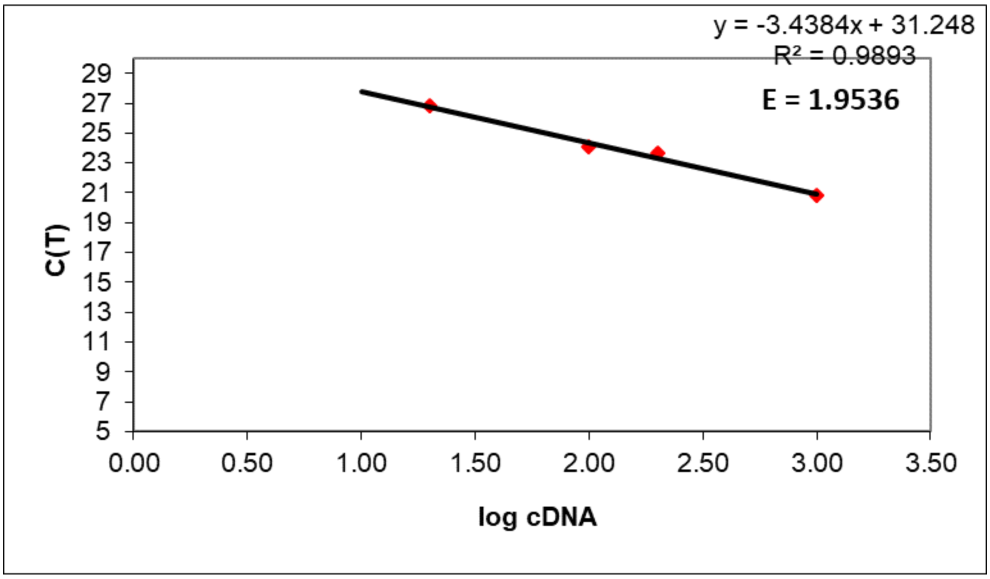
y = -3.4384x + 31.248
R² = 0.9893
E = 1.9536
C(T)
29
27
25
23
21
19
17
15
13
11
9
7
5
0.00
0.50
1.00
1.50
2.00
2.50
3.00
3.50
log cDNA

O)

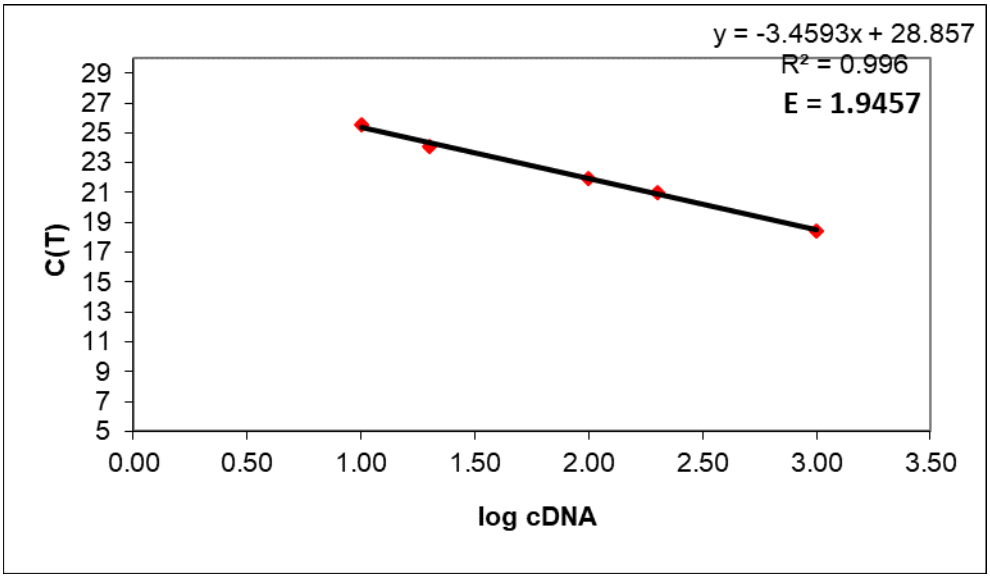
y = -3.4593x + 28.857
R² = 0.996
E = 1.9457
C(T)
29
27
25
23
21
19
17
15
13
11
9
7
5
0.00
0.50
1.00
1.50
2.00
2.50
3.00
3.50
log cDNA

References

1. Floyd, E.E.; Gong, Z.; Brandhorst, B.P.; Klein, W.H. *Calmodulin* gene expression during sea urchin development: Persistence of a prevalent maternal protein. *Dev. Biol.* **1986**, *113*, 501–511.

2. Baitinger, C.; Alderton, J.; Poenie, M.; Schulman, H.; Steinhardt, R.A. Multifunctional Ca2+/calmodulin-dependent protein kinase is necessary for nuclear envelope breakdown. *J. Cell Biol.* **1990**, *111*, 1763–1773.

3. Ingham, P.W. Transducing Hedgehog: The story so far. *EMBO J.* **1998**, *17*, 3505–3511.

4. Cunha, I.; García, L.M.; Guilhermino, L. Sea-urchin (*Paracentrotus lividus*) glutathione S-transferases and cholinesterase activities as biomarkers of environmental contamination. *J. Environ. Monit.* **2005**, *7*, 288–294.

5. Gambardella, C.; Aluigi, M.G.; Ferrando, S.; Gallus, L.; Ramoino, P.; Gatti, A.M.; Rottigni, M.; Falugi, C. Developmental abnormalities and changes in cholinesterase activity in sea urchin embryos and larvae from sperm exposed to engineered nanoparticles. *Aquat. Toxicol.* **2013**, *130–131*, 77–85.

6. Goldstone, J. V.; Hamdoun, A.; Cole, B.J.; Howard-Ashby, M.; Nebert, D.W.; Scally, M.; Dean, M.; Epel, D.; Hahn, M.E.; Stegeman, J.J. The chemical defensome: Environmental sensing and response genes in the *Strongylocentrotus purpuratus* genome. *Dev. Biol.* **2006**, *300*, 366–384.

7. Goldstone, J. V.; Goldstone, H.M.H.; Morrison, A.M.; Tarrant, A.; Kern, S.E.; Woodin, B.R.; Stegeman, J.J. Cytochrome P450 1 genes in early deuterostomes (tunicates and sea urchins) and vertebrates (chicken and frog): Origin and diversification of the *CYP1* gene family. *Mol. Biol. Evol.* **2007**, *24*, 2619–2631.

8. Hursh, D.A.; Andrews, M.E.; Raff, R.A. A sea urchin gene encodes a polypeptide homologous to epidermal growth factor. *Science (80-.).* **1987**, *237*, 1487–1490.

9. Bisgrove, B.W.; Andrews, M.E.; Raff, R.A. Fibropellins, DONT USE. *Dev. Biol.* **1991**, *146*, 89–99.

10. Yamasu, K.; Suzuki, G.; Horii, K.; Suyemitsu, T. Transcriptional regulation of the gene for epidermal growth factor-like peptides in sea urchin embryos. *Int. J. Dev. Biol.* **2000**, *44*, 777–784.

11. Lhomond, G.; McClay, D.R.; Gache, C.; Croce, J.C. *Frizzled1/2/7* signaling directs β-catenin nuclearisation and initiates endoderm specification in macromeres during sea urchin embryogenesis. *Development* **2012**, *139*, 816–825.

12. Mita, M.; Yasumasu, I. Metabolism of lipid and carbohydrate in sea urchin spermatozoa. *Gamete Res.* **1983**, *7*, 133–144.

13. Miki, K.; Qu, W.; Goulding, E.H.; Willis, W.D.; Bunch, D.O.; Strader, L.F.; Perreault, S.D.; Eddy, E.M.; Brien, D.A.O. a Sperm-Specific Glycolytic Enzyme , Is Required for Sperm Motility and Male Fertility. *Sci. York* **2004**, *101*.

14. Muronetz, V.I.; Kuravsky, M.L.; Barinova, K. V.; Schmalhausen, E. V. Sperm-Specific *Glyceraldehyde-3-Phosphate Dehydrogenase*-An Evolutionary Acquisition of Mammals. *Biochem.* **2015**, *80*, 1672–1689.

15. Sconzo, G.; Amore, G.; Capra, G.; Giudice, G.; Cascino, D.; Ghersi, G. Identification and characterization of a constitutive HSP75 in sea urchin embryos. *Biochem. Biophys. Res. Commun.* **1997**, *234*, 24–29.

16. Geraci, F.; Pinsino, A.; Turturici, G.; Savona, R.; Giudice, G.; Sconzo, G. Nickel, lead, and cadmium induce differential cellular responses in sea urchin embryos by activating the synthesis of different HSP70s. *Biochem. Biophys. Res. Commun.* **2004**, *322*, 873–877.

17. Bédard, P.A.; Brandhorst, B.P. Translational activation of maternal mRNA encoding the heat-shock

protein hsp90 during sea urchin embryogenesis. *Dev. Biol.* **1986**, *117*, 286–293.

18. Walton, Katherine C., Warner, Jacob,Hertzler, Philip H., McClay, D.R. *Hedgehog* signaling patterns mesoderm in the sea urchin. *Dev. Biol.* **2009**, *331*, 26–37.

19. Hou, S.X.; Zheng, Z.; Chen, X.; Perrimon, N. The *JAK/STAT* pathway in model organisms: Emerging roles in cell movement. *Dev. Cell* **2002**, *3*, 765–778.

20. Ito, M.; Nakasato, M.; Suzuki, T.; Sakai, S.; Nagata, M.; Aoki, F. Localization of Janus kinase 2 to the nuclei of mature oocytes and early cleavage stage mouse embryos. *Biol. Reprod.* **2004**, *71*, 89–96.

21. Warner, J.F.; Mcclay, D.R. Left-right asymmetry in the Sea Urchin. *Genesis* **2014**, *52*, 481–487.

22. Range, R.; Lepage, T. Maternal *Oct1/2* is required for Nodal and *Vg1/Univin* expression during dorsal-ventral axis specification in the sea urchin embryo. *Dev. Biol.* **2011**, *357*, 440–449.

23. Fujiwara, Akiko, Yasumasu, I. Does the respiratory rate in sea urchin embryos increase during early development without proliferation of mitochondria? *Dev. growth fifferentation* **1997**, *39*, 179–189.

24. Loza-Huerta, A.; Vera-Estrella, R.; Darszon, A.; Beltrán, C. Certain *Strongylocentrotus purpuratus* sperm mitochondrial proteins co-purify with low density detergent-insoluble membranes and are PKA or PKC-substrates possibly involved in sperm motility regulation. *Biochim. Biophys. Acta - Gen. Subj.* **2013**, *1830*, 5305–5315.

25. Röttinger, E.; Croce, J.; Lhomond, G.; Besnardeau, L.; Gache, C.; Lepage, T. *Nemo-like kinase* (*NLK*) acts downstream of *Notch/Delta* signalling to downregulate *TCF* during mesoderm induction in the sea urchin embryo. *Development* **2006**, *133*, 4341–4353.

26. Royet, J.; Bouwmeester, T.; Cohen, S.M. *Notchless* encodes a novel WD40-repeat-containing protein that modulates notch signaling activity. *EMBO J.* **1998**, *17*, 7351–7360.

27. Lossie, A.C.; Lo, C.L.; Baumgarner, K.M.; Cramer, M.J.; Garner, J.P.; Justice, M.J. ENU mutagenesis reveals that *Notchless homolog 1* (*Drosophila*) affects *Cdkn1a* and several members of the *Wnt* pathway during murine pre-implantation development. *BMC Genet.* **2012**, *13*.

28. Coward, K.; Owen, H.; Poustka, A.J.; Hibbitt, O.; Tunwell, R.; Kubota, H.; Swann, K.; Parrington, J. Cloning of a novel *phospholipase C-δ isoform* from pacific purple sea urchin (*Strongylocentrotus purpuratus*) gametes and its expression during early embryonic development. *Biochem. Biophys. Res. Commun.* **2004**, *313*, 894–901.

29. Sidhu, R.S.; Clough, R.R.; Bhullar, R.P. Regulation of *phospholipase C-δ1* through direct interactions with the small *GTPase Ral* and *calmodulin*. *J. Biol. Chem.* **2005**, *280*, 21933–21941.

30. Pulcrano, G.; Leonardo, R.; Aniello, F.; Mancini, P.; Piscopo, M.; Branno, M.; Fucci, L. *PLAUF* is a novel *P. lividus* sea urchin RNA-binding protein. *Gene* **2005**, *347*, 99–107.

31. Beeble, A.; Calestani, C. Expression pattern of *polyketide synthase-2* during sea urchin development. *Gene Expr. Patterns* **2012**, *12*, 7–10.

32. Nachtergaele, S.; Whalen, D.M.; Mydock, L.K.; Zhao, Z.; Malinauskas, T.; Krishnan, K.; Ingham, P.W.; Covey, D.F.; Siebold, C.; Rohatgi, R. Structure and function of the *Smoothened* extracellular domain in vertebrate *Hedgehog* signaling. *Elife* **2013**, *2*, 1–32.

33. Brown, S.; Hu, N.; Hombría, J.C.G. Identification of the first invertebrate interleukin *JAK/STAT* receptor, the *Drosophila* gene domeless. *Curr. Biol.* **2001**, *11*, 1700–1705.

34. Darnell, J.E. STATs and gene regulation. *Science (80-.).* **1997**, *277*, 1630–1635.

35. Barrett, Kathleen G., Fang, Hailin, Kocarek, Thomas A., Runge-Morris, M. Transcriptional Regulation

of *SULT1C2* by Vitamin D Receptor in LS180 Human Colorectal Adenocarcinoma Cells. *Drug Metab. Dispos.* **2016**, *42*, 361–368.

36. Vereecke, L.; Beyaert, R.; Van Loo, G. Genetic relationships between A20/TNFAIP3, chronic inflammation and autoimmune disease. *Biochem. Soc. Trans.* **2011**, *39*, 1086–1091.

37. Esposito, R.; Ruocco, N.; Albarano, L.; Ianora, A.; Manfra, L.; Libralato, G.; Costantini, M. Combined effects of diatom-derived oxylipins on the sea urchin *Paracentrotus lividus*. *Int. J. Mol. Sci.* **2020**, *21*, 24–27.

38. Romano, G.; Costantini, M.; Buttino, I.; Ianora, A.; Palumbo, A. Nitric oxide mediates the stress response induced by diatom aldehydes in the sea urchin *Paracentrotus lividus*. *PLoS One* **2011**, *6*, 2–10.

39. Varrella, S.; Romano, G.; Costantini, S.; Ruocco, N.; Ianora, A.; Bentley, M.G.; Costantini, M. Toxic diatom aldehydes affect defence gene networks in sea urchins. *PLoS One* **2016**, *11*, 1–21.

40. Marrone, V.; Piscopo, M.; Romano, G.; Ianora, A.; Palumbo, A.; Costantini, M. Defensome against toxic diatom aldehydes in the sea urchin *Paracentrotus lividus*. *PLoS One* **2012**, *7*, 1–8.

41. Ruocco, N.; Cavaccini, V.; Caramiello, D.; Ianora, A.; Fontana, A.; Zupo, V.; Costantini, M. Noxious effects of the benthic diatoms *Cocconeis scutellum* and *Diploneis sp.* on sea urchin development: Morphological and de novo transcriptomic analysis. *Harmful Algae* **2019**, *86*, 64–73.

42. Russo, R.; Pinsino, A.; Costa, C.; Bonaventura, R.; Matranga, V.; Zito, F. The newly characterized Pl-jun is specifically expressed in skeletogenic cells of the *Paracentrotus lividus* sea urchin embryo. *FEBS J.* **2014**, *281*, 3828–3843.

43. Varrella, S.; Romano, G.; Ianora, A.; Bentley, M.G.; Ruocco, N.; Costantini, M. Molecular response to toxic diatom-derived aldehydes in the sea urchin *Paracentrotus lividus*. *Mar. Drugs* **2014**, *12*, 2089–2113.

44. Ruocco, N.; Maria Fedele, A.; Costantini, S.; Romano, G.; Ianora, A.; Costantini, M. New inter-correlated genes targeted by diatom-derived polyunsaturated aldehydes in the sea urchin *Paracentrotus lividus*. *Ecotoxicol. Environ. Saf.* **2017**, *142*, 355–362.

45. Costa, C.; Karakostis, K.; Zito, F.; Matranga, V. Phylogenetic analysis and expression patterns of *p16* and p19 in *Paracentrotus lividus* embryos. *Dev. Genes Evol.* **2012**, *222*, 245–251.

46. Ragusa, Maria Antonietta, Costa , Salvatore, Gianguzza, Marco, Roccheri, Maria Carmela, Gianguzza, F. Effects of cadmium exposure on sea urchin development assessed by SSH and *RT-qPCR* : metallothionein genes and their differential induction. *Mol. Biol. Rep.* **2013**, *40*, 2157–2167.
